# Structural integrity of the substantia nigra and subthalamic nucleus predicts flexibility of instrumental learning in older-age individuals

**DOI:** 10.1016/j.neurobiolaging.2013.03.030

**Published:** 2013-10

**Authors:** Rumana Chowdhury, Marc Guitart-Masip, Christian Lambert, Raymond J. Dolan, Emrah Düzel

**Affiliations:** a Institute of Cognitive Neuroscience, University College London, London, UK; b Wellcome Trust Centre for Neuroimaging, Institute of Neurology, University College London, London, UK; c Institute of Cognitive Neurology and Dementia Research, Otto-von-Guericke-University Magdeburg, Magdeburg, Germany; d German Center for Neurodegenerative Diseases (DZNE), Magdeburg, Germany

**Keywords:** Aging, Instrumental learning, Magnetization transfer, Novelty seeking, Substantia nigra

## Abstract

Flexible instrumental learning is required to harness the appropriate behaviors to obtain rewards and to avoid punishments. The precise contribution of dopaminergic midbrain regions (substantia nigra/ventral tegmental area [SN/VTA]) to this form of behavioral adaptation remains unclear. Normal aging is associated with a variable loss of dopamine neurons in the SN/VTA. We therefore tested the relationship between flexible instrumental learning and midbrain structural integrity. We compared task performance on a probabilistic monetary go/no-go task, involving trial and error learning of: “go to win,” “no-go to win,” “go to avoid losing,” and “no-go to avoid losing” in 42 healthy older adults to previous behavioral data from 47 younger adults. Quantitative structural magnetization transfer images were obtained to index regional structural integrity. On average, both some younger and some older participants demonstrated a behavioral asymmetry whereby they were better at learning to act for reward (“go to win” > “no-go to win”), but better at learning not to act to avoid punishment (“no-go to avoid losing” > “go to avoid losing”). Older, but not younger, participants with greater structural integrity of the SN/VTA and the adjacent subthalamic nucleus could overcome this asymmetry. We show that interindividual variability among healthy older adults of the structural integrity within the SN/VTA and subthalamic nucleus relates to effective acquisition of competing instrumental responses.

## 1 Introduction

To efficiently harvest reward and avoid punishment, humans need to learn appropriate instrumental responses (Dickinson and Balleine, 2002) (O'Doherty et al., 2004). Recent data suggest that this basic form of behavioral adaptation is surprisingly inflexible in humans (Guitart-Masip et al., 2012b). Although healthy young human adults readily learn to act to obtain a reward, or not to act to avoid a punishment, they have difficulties learning to act to avoid a punishment and not to act to obtain a reward (Guitart-Masip et al., 2012b). This inflexibility in learning suggests that signals that predict rewards are prepotently associated with behavioral activation promoting approach behavior, whereas signals associated with punishments are intrinsically coupled to behavioral inhibition promoting avoidance. These behavioral tendencies can be described as Pavlovian biases that corrupt the flexibility of instrumental learning (Dayan et al., 2006; Gray and McNaughton, 2000). Computational modeling in young adults has shown that the observed pattern of behavior is captured by a model incorporating a Pavlovian bias, where the strength of this bias is related to failure to learn the conflicting conditions: no-go to win and go to avoid losing (Guitart-Masip et al., 2012b).

The substantia nigra/ventral tegmental area (SN/VTA) of the midbrain, the origin of dopaminergic projections, is important for instrumental learning (Salamone et al., 2005; Schultz et al., 1997) including signaling reward predictions errors (Schultz et al., 1997), energizing actions (Niv et al., 2007), and driving novelty-related exploratory behavior (Duzel et al., 2010; Lisman et al., 2011). In humans, dopaminergic medication after learning influences the brain responses to action and to reward anticipation (Guitart-Masip et al., 2012a). Importantly, the SN/VTA undergoes degeneration with aging (Bäckman et al., 2006; Fearnley and Lees, 1991; Vaillancourt et al., 2012). Age differences in instrumental learning have been linked to functional activity in dopaminergic target regions, including the striatum and prefrontal cortex (Samanez-Larkin et al., 2010) (Mell et al., 2009) (Fera et al., 2005) (Aizenstein et al., 2006). Structural degeneration of the SN/VTA and associated circuits can be indexed in vivo by magnetization transfer (MT) imaging, in which lower MT values reflect decreased structural integrity (Düzel et al., 2008; Eckert et al., 2004; Tambasco et al., 2011).

The goal of this study was to relate individual differences of SN/VTA integrity in older age to flexible instrumental learning for competing responses (“to act” or “not to act”) to rewards and punishments. Furthermore, to explore age-group comparisons of learning and structural integrity of SN/VTA, we obtained separate data from younger adults. We hypothesized that older adults with higher SN/VTA integrity would show greater learning flexibility. Thus, instrumentally learning to act to avoid a punishment and not to act to obtain a reward would be equivalent to learning to act to obtain a reward or not to act to avoid a punishment, the latter being Pavlovian response biases that tend to dominate learning. We also obtained trait measures of novelty seeking in older adults to test the relationship with instrumental learning and structural integrity.

## 2 Methods

### 2.1 Participants

#### 2.1.1 Older participants

A total of 42 healthy older adults aged 64 to 75 years (mean = 69.12 years. SD = 3.44 years; 29 females and 13 male, 40 right-handed and 2 left-handed) were recruited via our departmental Web site, advertisement in local public buildings, and word of mouth. Individuals were initially screened by telephone and excluded if they had any of the following: current or past history of neurological, psychiatric, or endocrinological disorders; metallic implants, tinnitus; major visual impairment; or history of drug dependency. To control for vascular risk factors, individuals known to have had a stroke or transient ischemic attack, myocardial infarction or other significant cardiovascular history, diabetes mellitus or hypertension requiring more than 1 antihypertensive medication were not eligible for participation. All participants undertook a neuropsychological test battery to ensure intact global cognitive performance (Supplementary Table 1). On the basis of this, no participants were excluded from the analysis (all participants scored within 1.5 SDs of the age-related norm for each test). All participants had a normal neurological examination (performed by a physician [R.C.]) ensuring that participants did not have concurrent undiagnosed neurological conditions. MRI scans were visually inspected to ensure that no participants had severe white matter changes or other major lesions. Clinical examination, neuropsychological testing, the go/no-go task, and structural MRI scanning were all performed in a single 4-hour session. Written informed consent was obtained from all participants. The study received ethical approval from the North West London Research Ethics Committee 2.

#### 2.1.2 Younger participants

Data from 2 previously published experiments performed at the host institution were obtained to enable separate age-comparisons of behavioral data and MRI data. In 1 study, behavioral data from 47 healthy young adults (28 female and 19 male; mean age = 23.1 years, SD = 4.1 years) performing the same go/no-go task was obtained allowing comparisons of behavioral performance between young and older adults (Guitart-Masip, et al., 2012b). Structural neuroimaging including MT imaging was available for 30 of these younger adults, which we used to examine the correlation between SN/VTA integrity and task performance in younger adults. These scans were obtained on a different MRI scanner (3-T Siemens Allegra) using a different acquisition protocol that did not include B1 correction (Guitart-Masip, et al., 2012b for details), thus direct age-comparisons of actual MT values could not be made with this dataset and ours.

Therefore in the second study, neuroimaging data from 12 healthy younger adults (6 female and 6 male; mean age = 33.8 years, SD = 12.84 years), using the same MRI scanner and imaging sequence, was obtained to allow comparison of MT values of SN/VTA between younger and older adults (Lambert et al., 2012 for details).

### 2.2 Go/no-go task

**Fig. 1:**
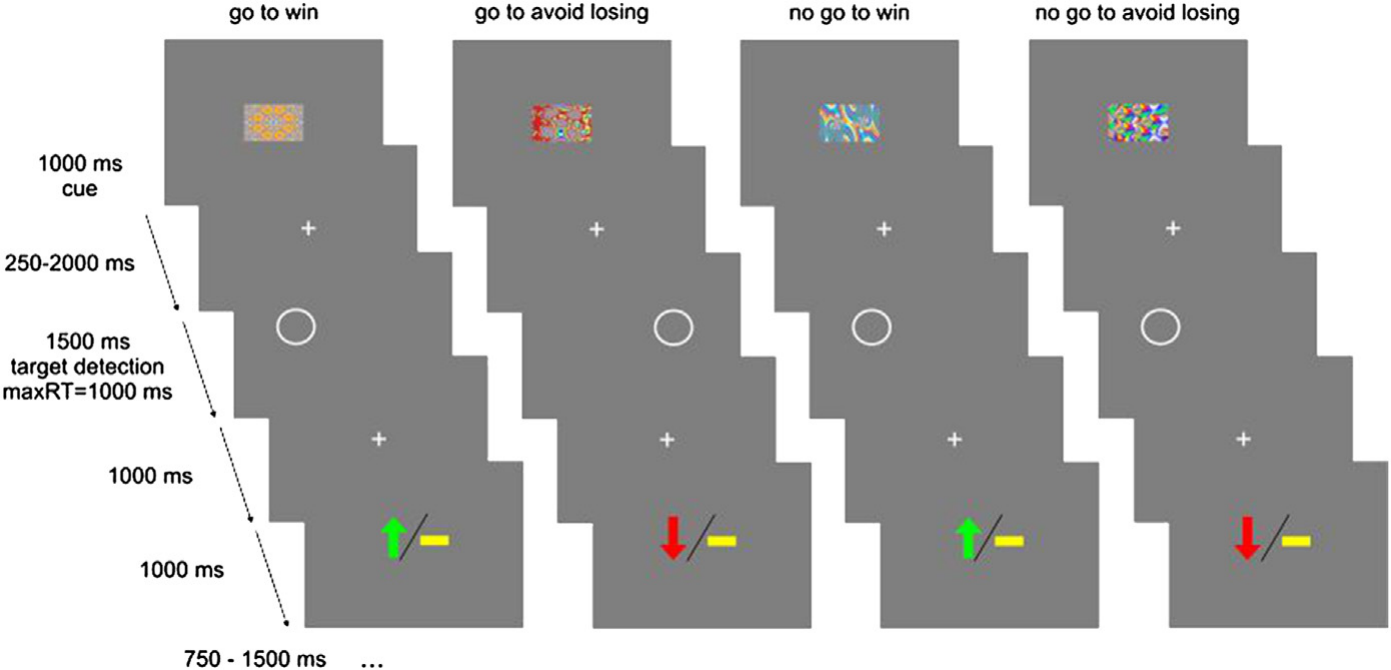
Probabilistic monetary go/no-go task.

Participants performed a probabilistic monetary go/no-go task as described in Guitart-Masip et al. (Guitart-Masip et al., 2012b) (Fig. 1). The correct response (to execute or withhold an action) to 4 cues (abstract fractal images) had to be learned through trial and error, to win or to avoid losing money. Participants were told that at the start of the task they would not know the correct responses (to press or not to press a button) for each image, but that these would become clear through trial and error. After seeing an image (1000 ms), there was a variable interval (250–2000 ms) after which participants were presented with a circle (target detection, 1500 ms), at which point they had to either press a button (go) with their dominant hand to indicate the target side within 1000 ms or not press a button (no-go). After this, the outcome was depicted for 1000 ms by a green up-pointing arrow (indicating a win of £1), a red down-pointing arrow (indicating a loss of £1), or a yellow horizontal bar (neither win nor lose). The outcome was probabilistic, whereby in the win conditions, 80% of correct choices and 20% of incorrect choices were rewarded (the remaining 20% of correct and 80% of incorrect choices leading to a neutral outcome). In the lose conditions, 80% of correct choices and 20% of incorrect choices avoided punishment (the remaining 20% of correct and 80% of incorrect choices leading to a neutral outcome). The probabilistic nature of the task was made clear to participants in the written and verbal instructions before the task. Thus, the task consisted of 4 trial types depending on the nature of the fractal cue presented at the beginning of the trials:

• Press the correct button in the target detection task to gain a reward (go to win [GW])
• Press the correct button in the target detection task to avoid punishment (go to avoid losing [GAL])
• Do not press a button in the target detection task to gain a reward (no-go to win [NGW])
• Do not press a button in the target detection task to avoid punishment (no-go to avoid losing [NGAL])

The task consisted of 240 trials (60 trials for each of the 4 conditions, presented in a randomized fashion) and lasted ∼35 minutes. At the beginning of the task, participants were told that they could win between £5 to £15 and were given their earnings on task completion. Before the actual task, participants undertook a brief training session of 10 practice trials in which only the target detection circles were presented. Participants were instructed to press the corresponding button for every target (left arrow key on the keyboard if the target appeared on the left of the screen and vice versa for the right). This allowed participants to familiarize themselves with the appropriate buttons on the computer keyboard and to obtain an overall feel for the speed of the task without exposure to any of the cues used in the main task.

### 2.3 Tridimensional Personality Questionnaire

Each participant completed the Tridimensional Personality Questionnaire (TPQ) (Cloninger 1987). This self-report questionnaire consists of 100 true-or-false items measuring 3 personality traits: novelty seeking, harm avoidance, and reward dependence.

### 2.4 MRI scanning

A high-resolution structural MRI dataset for each participant was obtained on a 3.0T MRI scanner (Magnetom TIM Trio, Siemens Healthcare, Erlangen, Germany) using a 32-channel head coil. A structural multi-parameter map protocol using a 3-dimensional (3D) multi-echo fast low angle shot (FLASH) sequence at 1 mm isotropic resolution was used to acquire MT weighted images (echo time, TE, 2.2–14.70 ms; repetition time, TR, 23.7 ms; flip angle, FA, 6°), proton density weighted (TE 2.2–19.7 ms, TR 23.7 ms, FA 6°) and T1 weighted (TE 2.2–14.7 ms, TR 18.7 ms, FA 20°) (Helms et al., 2008). B1 mapping (TE 37.06 and 55.59 ms, TR 500 ms, FA 230:−10:130 degrees, 4 mm^3^ isotropic resolution) was acquired to correct the T1 maps for inhomogeneities in the transmit radiofrequency field (Lutti et al., 2010). A double-echo FLASH sequence (TE1 10 ms, TE2 12.46 ms, 3 × 3 × 2-mm resolution and 1-mm gap) was used to measure local field inhomogeneities and to correct for the image distortions in the B1 mapping data.

Using an in-house code, the MT, T1, and R2* (1/T2*) quantitative maps were extracted for each subject from the anatomical scans described above. Proton density scans were not used for any analyses but were acquired, as they are crucial for estimating MT and T1 parameters; for full details regarding the generation of quantitative maps see Helms et al. (Helms et al., 2008). MT, T1 and R2* values reflect structural integrity (Düzel et al., 2008; Eckert et al., 2004; Tambasco et al., 2011; Wolff and Balaban, 1989), myelin, and iron content, respectively (Draganski et al., 2011; Martin, 2009; Martin et al., 2008).

### 2.5 Imaging analysis

Data processing and analysis was performed using Statistical Parametric Mapping software (SPM8; Wellcome Trust Centre for Neuroimaging, London, UK) and MATLAB 7.8 (Mathworks, Sherborn, MA, USA). Two independent analyses were conducted with the structural MRI data. The first was a region-of-interest analysis (ROI) of the SN/VTA performed in both younger and older adults. The second was a whole-brain voxel-based analysis performed in older adults only.

### 2.6 Definition of ROIs

#### 2.6.1 Substantia nigra/ventral tegmental area

**Supplementary Fig. 1:**
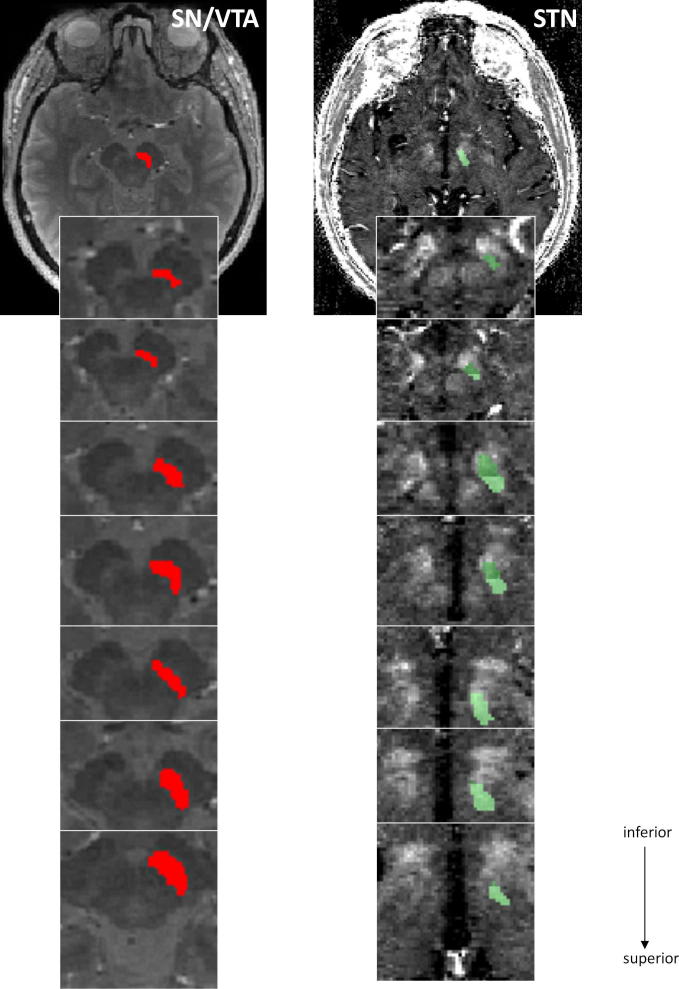


The medial and lateral boundaries of the substantia nigra/ventral tegmental area (SN/VTA) were defined on each participant's MT-weighted image, where it was easily distinguishable from the surrounding tissues because of its bright gray color in contrast to the adjacent cerebral peduncle. For each subject, this region was manually defined on every visible slice, usually between 7 to 10 slices as per Düzel et al (Düzel et al., 2008) using MRIcro (Rorden, 2000). A single-slice example from a single subject is shown in Fig. 3A (Supplementary Fig. 1 is an example of all slices from a single subject). For all subjects, their ROIs were projected as an overlay on their MT, T1, and R2* maps to obtain a mean value for the region. Bilateral SN/VTA values in older adults, calculated by averaging right and left SN/VTA values, were as follows (in arbitrary units): MT mean = 0.93 (SD = 0.070), T1 mean = 1129.31 (SD = 51.23), and R2* mean = 0.028 (SD = 0.0048).

#### 2.6.2 Subthalamic nucleus

The subthalamic nucleus (STN) was manually segmented for each subject using the software package ITK-SNAP (Yushkevich et al., 2006) as described in Lambert et al. (Lambert et al., 2012). Briefly, using R2* maps, it appears as a hyperintense region. The borders of the STN were defined as the zona incerta superiorly and immediately medially; preleminiscal radiations, posterior–lateral hypothalamus, and red nucleus further medially and cerebral peduncle laterally. The inferior tip lies on the superior aspect of the substantia nigra at the level of the optic tract. Supplementary Fig. 1 provides a single-subject example.

Ten randomly selected SN/VTA and STN ROIs were segmented by a second trained individual (C.L. and R.C., respectively), showing high interrater reliability (SN/VTA: intraclass correlation = 0.87, *p* < 0.0005; STN: intraclass correlation = 0.98, *p* < 0.0005).

### 2.7 Magnetization transfer subgroups

We obtained magnetization transfer (MT) data for 12 younger adults (mean age = 33.8 years, SD = 12.84; 6 female and 6 male) from a separate published experiment (Lambert et al., 2012). For comparison, we formed 2 subgroups each consisting of 12 older adults matched for age and gender (10 females and 2 males per group), that differed significantly in MT values of the right SN/VTA (independent-samples t test, 2-tailed: t(22) = −9.93, *p* < 0.0001). For these subgroups, 12 older adults with the highest and lowest MT values of the right SN/VTA were selected to form a “high MT” group (MT: mean = 0.98, SD = 0.038; mean age = 69.33 years, SD = 2.74 years) and “low MT” group respectively (MT: mean = 0.84, SD = 0.023; mean age = 70.08 years, SD = 3.34 years). We used right SN/VTA for post hoc tests of the MT subgroups based on the major VBQ finding of a correlation between right SN/VTA integrity and NGW performance. We used these subgroups for 3 analyses: first, to further explore the relationship between MT and behavior within older adults only; second, to compare MT values of the SN/VTA between younger and older adults; and third, to compare novelty-seeking scores within older adults only.

### 2.8 Voxel-based quantification

To explore the regional specificity of the correlation between SN/VTA integrity and task performance in older adults, a method recently termed voxel-based quantification (VBQ) was used (Draganski et al., 2011). This allows whole-brain statistical analysis of quantitative MRI parameters such as MT. The methodology was adapted from Draganski et al. (2011) with a few adjustments specific to the current cohort summarized as follows. In brief, unified segmentation was used to classify MT maps into gray matter, white matter, and cerebrospinal fluid (Ashburner and Friston, 2005). Although better segmentation of subcortical regions can be attained using MT rather than T1 maps (Helms et al., 2009), visual inspection revealed that the SN/VTA region was often incomplete and misclassified as white matter. Therefore, in subject space, the manually defined SN/VTA ROI was added to each unmodulated gray matter mask and subtracted from the white matter. These maps were adjusted to ensure that all voxels remained in the range from 0 to 1. Using a diffeomorphic registration algorithm (DARTEL), the MT white and gray matter maps were warped to a common template (Ashburner, 2007). Modulation was achieved by multiplying these warped images with their Jacobian determinants. Finally, weighted-average MT maps were created as previously described (Draganski et al., 2011) and were smoothed with an isotropic Gaussian kernel of 6 mm full width at half maximum.

### 2.9 Statistical analysis

Performance in each of the 4 task conditions was calculated as the percentage of correct responses and analyzed using a repeated-measures analysis of variance (ANOVA) with action (go/no-go) and valence (win/avoid loss) as the within-subjects factors. To compare performance between older MT subgroups, MT-group (low/high) was added as a between-subjects factor. To compare performance between all young and all older adults, age-group (young/older) was added as a between-subjects factor. To further explore behavioral response biases in go/no-go task performance in older age, we calculated the following measures using the total number of correct trials per condition: main effect of action (GW+GAL-NGW-NGAL), main effect of valence (GW+NGW-GAL-NGAL), and an interaction between action and valence (GW+NGAL-NGW-GAL). Partial Pearson's correlations (controlling for age and SN/VTA volume) were used to correlate response biases with SN/VTA MT values (significance level set at *p* < 0.017 after Bonferroni correction for 3 tests) and to assess the relationship between the behavioral interaction and personality measures of novelty seeking, reward dependence, and harm avoidance (significance level set at *p* < 0.017 after Bonferroni correction for 3 tests). All reported significance values are 2-tailed.

For structural imaging parameters of SN/VTA, linear multiple regression analyses were performed using the Statistical Package for the Social Sciences (SPSS), version 17.0. We used a backward model to conduct a separate analysis for each of the 4 task conditions (GW, GAL, NGW, NGAL) where performance (percentage of total correct responses) in these conditions was used as the dependent variable. The 5 independent variables in each model were the 3 imaging parameter values of bilateral SN/VTA (MT, T1, and R2* values), volume of the SN/VTA and age. The significance level for each model was set at *p* < 0.0125 (Bonferroni correction for 4 models). To address co-variance between MT and T1 values (Supplementary Table 2), we also report separate correlations between neuroimaging parameters and task performance (Supplementary Table 3). All reported significance values are 2-tailed.

The VBQ analysis was performed only for the significant task condition (NGW) and image type (MT) from the behavioral regression analyses to minimize the number of voxel-based analyses. The calculated weighted-average MT maps were analyzed in a multiple regression model in SPM8. A single analysis was performed using a design matrix containing performance in all 4 task conditions (GW, GAL, NGW, NGAL) as separate covariates and age, gender, and total intracranial volume (sum of gray matter, white matter, and CSF) as regressors of no interest. We included performance in all 4 task conditions in a single model as a more stringent test to identify the unique variance associated with NGW performance over and above performance in the other conditions (Supplementary Table 4 shows no significant covariance between these measures). An explicit mask created from the gray matter probability maps thresholded at 0.2 was applied. Uncorrected whole-brain *p* values were <0.001 for clusters greater than 10 voxels are reported. We created SN/VTA and STN masks for small-volume correction using individual participants' manually defined ROIs, normalized to MNI space using DARTEL and group averaged. A statistical threshold of *p* < 0.05 after familywise error correction was used for the hypothesis-based small-volume correction analyses.

## 3 Results

### 3.1 Go/no-go task performance in older adults

**Fig. 2:**
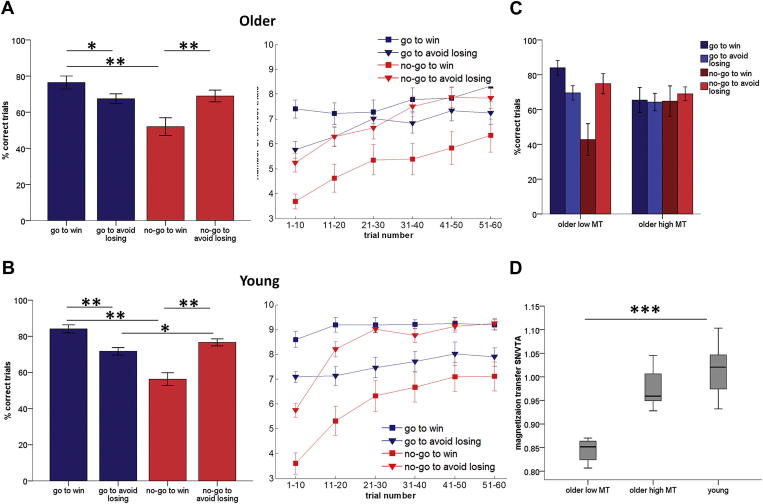
Go/no-go task performance in older and younger adults. (A, left) Older participants (n = 42) had an asymmetry in action–valence learning, such that they were better at learning active choices for a reward (“go to win”) than to avoid punishment (“go to avoid losing”), whereas they were better at learning passive choices to avoid punishment (“no-go to avoid losing”) than for reward (“no-go to win”). (A, right) Older adults began the task with a bias toward choosing an action (“go”). Learning occurred in all conditions over the course of the task. (B, left and right) A similar overall pattern of behavior was evident in 47 younger adults. (C) A “high-MT” subgroup of 12 older individuals with higher substantia nigra/ventral tegmental area (SN/VTA) integrity (high magnetization transfer [MT]) could overcome response biases to acquire competing responses for reward, compared to a subgroup of 12 older adults with lower SN/VTA integrity (“low MT”). (D) This “low MT” subgroup of older adults (n = 12) had significantly lower MT values of SN/VTA than did 12 younger adults, whereas the “high MT” subgroup of older adults (n = 12) had similar MT values to those of the younger adults. Note that the younger group here is a different set of participants from those whose behavior is shown in B. Error bars represent ±1 SEM. * 0.01 < *p* < 0.05, ** *p* < 0.01, *** *p* < 0.0005.

Older participants were, on average, more accurate at go choices when the outcome was a reward (GW) and at no-go choices when the outcome was avoidance of losses (NGAL) [2 (go/no-go] by 2 (win/avoid loss) repeated-measures ANOVA: action–valence interaction: F_1,41_ = 12.55, *p* = 0.001; GW versus GAL: t(41) = 2.26, *p* = 0.029; NGW versus NGAL: t(41) = −3.20, *p* = 0.003; Fig. 2A). We also found a main effect of action indicating that participants were better at learning go compared to no-go choices (F_1,41_ = 7.29, *p* = 0.01). There was no main effect of valence (F_1,41_ = 1.87, *p* = 0.18). These results demonstrate that older adults had a marked asymmetry in their learning behavior.

Older adults showed a preponderant initial bias toward go responses (Fig. 2A) (1-sample t-test for performance in the first 10 trials: GW t(41) = 6.578, *p* = 0.000; GAL t(41) = 2.249, *p* = 0.030). In contrast, performance in the first 10 trials was at chance for the NGAL condition: [t(41) = 0.638, *p* = 0.527] and significantly below chance for NGW [t(41) = −4.365, *p* = 0.000]. This suggests a persisting action bias in the reward condition, whereas with loss a bias toward no-go responses emerged during learning. Over the course of the task, learning occurred in all conditions (Supplementary Table 5).

### 3.2 Structural neuroimaging in older adults

#### 3.2.1 ROI analysis

**Fig. 3:**
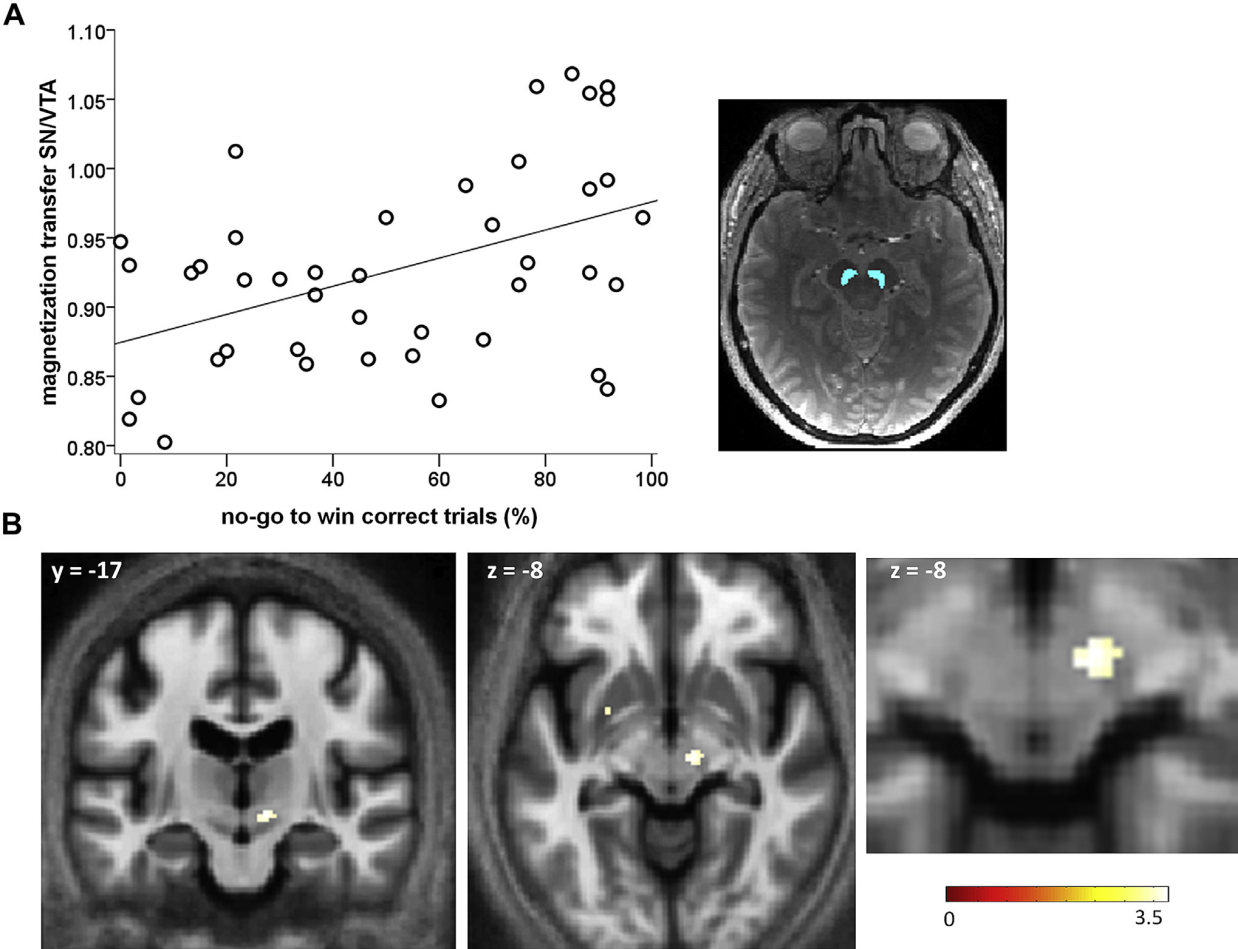
Higher no-go to win performance is associated with higher structural integrity of substantia nigra/ventral tegmental area (SN/VTA) and STN. (A) Region-of-interest analysis of the SN/VTA (single-subject, single-slice illustration of the bilateral SN/VTA region of interest (ROI), blue; see Supplementary Fig. 1 also). Scatter plot (in which each dot represents an individual) shows older individuals with higher SN/VTA integrity, indexed by higher magnetization transfer (MT) values, performed better in the no-go to win condition of the task. (B) An independent whole-brain voxel-based analysis of MT maps confirmed the association between higher MT values and no-go to win learning, localizing to a region overlapping with the right SN/VTA and right STN. Displayed on group-averaged MT image, uncorrected threshold *p* < 0.001.

For each experimental condition among older adults, we constructed a multiple regression model with task performance as the dependent variable and SN/VTA imaging parameter values (MT, T1, R2*), age, and SN/VTA volume as independent variables. These models explained only the variance in NGW performance in which the best model contained MT as the only explanatory variable (standardized β = 0.46, *p* = 0.002, R^2^ = 0.21). The additional variables did not add explanatory power (Table 1). Thus, higher SN/VTA integrity predicted an ability to learn to inhibit an action to obtain reward. Fig. 3A shows this correlation, which remained significant after controlling for both total intracranial volume and size of the SN/VTA (partial Pearson's r = 0.39, *p* = 0.014). Regression models for the remaining task conditions were not significant, suggesting that structural integrity, iron, or myelin content of SN/VTA were associated with learning the GW, GAL, or NGAL conditions (Supplementary Table 6).

**Fig. 4:**
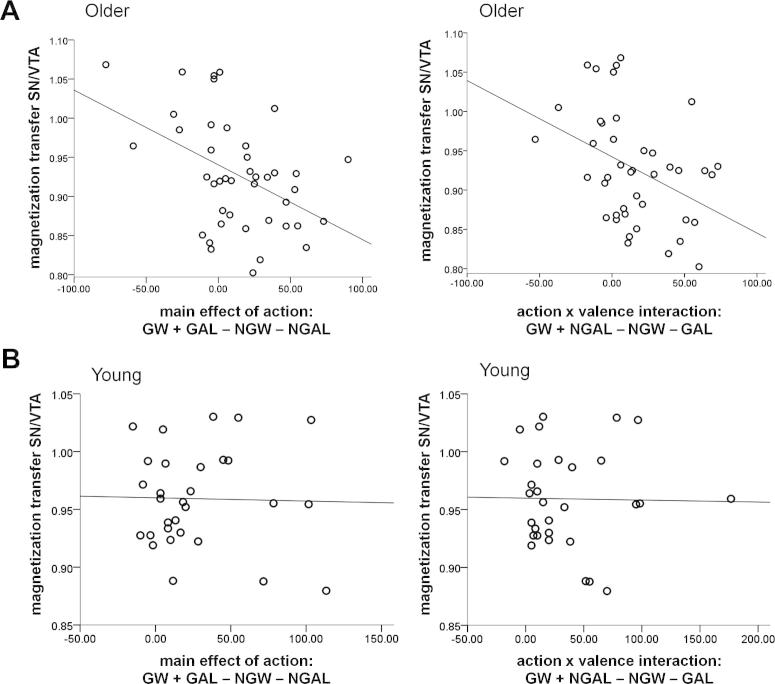
Relationship between substantia nigra/ventral tegmental area (SN/VTA) structural integrity and flexibility of instrumental learning. (A) Higher SN/VTA integrity (indexed by magnetization transfer values of SN/VTA) in older adults correlated with both a reduced action bias and reduced interaction between action and valence learning (n = 42). (B) No correlation between SN/VTA integrity and the action bias or the action–valence interaction in younger adults (n = 30). Scatter plots, in which each dot represents an individual. Abbreviations: GAL, go to avoid losing; GW, go to win; NGAL, no-go to avoid losing; NGW, no-go to win.

We next analyzed how SN/VTA integrity related to the ability to overcome response biases. The action bias (go > no-go performance for both wins and losses) was negatively correlated with SN/VTA integrity (r = −0.45, *p* = 0.003, Fig. 4A; partial correlation controlling for age and SN/VTA volume: r = −0.40, *p* = 0.011), suggesting that only those individuals with high SN/VTA integrity were able to overcome this action bias. Moreover, the negative correlation between the interaction in task performance (go to win and no-go to avoid losing > no-go to win and go to avoid losing performance) and SN/VTA integrity suggests the action–valence learning asymmetry could also be overcome with higher SN/VTA integrity (r = −0.42, *p* = 0.006, Fig. 4A; partial correlation controlling for age and SN/VTA volume: r =−0.35, *p* = 0.028). There was no correlation between SN/TA integrity and the main effect of valence (r = 0.22, *p* = 0.155; partial correlation controlling for age and SN/VTA volume: r = 0.27, *p* = 0.091). We found no evidence that working memory capacity contributed to the relationship between SN/VTA integrity and the pattern of task performance among older adults (Supplementary Results).

These results were also reflected in the older adult MT subgroup analyses, whereby we formed 2 gender-matched groups of older adults with the highest and lowest MT values of SN/VTA. We performed a repeated-measures ANOVA as before, with action (go/no-go) and valence (win/avoid loss) as within-subject factors but also included MT group (low/ high) as a between-subjects factor. Fig. 2C shows that the striking behavioral asymmetry between action and valence learning was present in the low MT group but not in the high MT group (three-way action by valence by MT-group interaction: F_1,22_ = 5.25, *p* = 0.032). Older individuals with low SN/VTA integrity were inflexible in learning the reward conditions: they readily learned the GW condition but were less able to concurrently learn the NGW condition. In contrast, older individuals with high SN/TA integrity were instrumentally more flexible, i.e., acquired both go and no-go responses concurrently to obtain rewards. However, higher flexibility in the high MT group came at a cost for GW performance with a trend towards a negative correlation between GW and NGW performance (r = −0.28, *p* =0.071) but not between GAL and NGAL (r = 0.13, *p* = 0.42). This suggests a trade-off between the ability to learn competing responses in the reward conditions. Similar to the assessment of behavior across all 42 older adults, this analysis of the MT-subgroups also demonstrated a trend towards a main effect of action (F_1,22_ = 3.16, *p* = 0.089), a significant action by valence interaction (F_1,22_ = 8.28, *p* = 0.009) and no main effect of valence (F_1,22_ = 1.47, *p* = 0.24). Overall, these results suggest that among older individuals, those with higher integrity of the SN/VTA were able to overcome their initial response biases, leading to more flexible instrumental learning, as evidenced by a more even performance across the different action–valence contingencies. Because the behavioral interaction was mostly driven by GW and NGW learning, these correlations also show that higher SN/VTA integrity confers flexibility by an improvement in NGW learning but with a concurrent slight decline in GW learning.

#### 3.2.2 Voxel-based quantification

To address potential bias from an ROI analysis, and to assess the anatomical specificity in the relationship between NGW performance and SN/VTA, we used a whole-brain voxel-based quantification (VBQ) analysis. This showed that positive correlations between NGW performance and MT values were restricted to a region that included the right SN/VTA and STN (Fig. 3B), with smaller clusters in the left cerebellum and left putamen only (Table 2).

For the SN/VTA and STN cluster, we quantified the percentage of overlap with probability maps of each anatomical region and found that 17.4% of the cluster overlapped with the STN, compared to 47.6% overlap with the SN/VTA. Using these probability maps, the multiple regression VBQ analysis of NGW performance and MT values of the right SN/VTA survived a hypothesis-based small volume correction (*p* < 0.05, FWE-corrected, Z_max_ = 3.39, x = 9, y = −17, z = −8). The same was true for the right STN (*p* < 0.05, FWE-corrected, Z_max_ = 3.33, x = 11, y = −17, z = −8).

### 3.3 Comparison of task performance between younger and older adults

To directly compare task performance between age groups, we obtained data from a separate experiment in which the same behavioral task was performed by 47 younger adults, of whom 30 underwent MT imaging (a detailed description of behavior among these younger adults can be found in Guitart-Masip et al., 2012b. A 2 × 2 repeated-measures ANOVA with action (go/no-go) and valence (win/avoid loss) as within-subject factors, and age group (young/older) as a between-subjects factor, showed a main effect of action (F_1,87_ = 21.75, *p* = 0.000), main effect of valence (F_1.87_ = 4.17, *p* = 0.044), and significant action by valence interaction (F_1,87_ = 47.23, *p* = 0.000), but no significant interaction of any factors with age group. Thus the overall pattern of performance showing a marked behavioral asymmetry was present in both younger and older adults (Fig. 2A and Fig. 2B). Performance averaged over all task conditions was worse in older adults (main effect of age F_1,88_ = 15.15, *p* < 0.0005).

**Supplementary Fig. 2:**
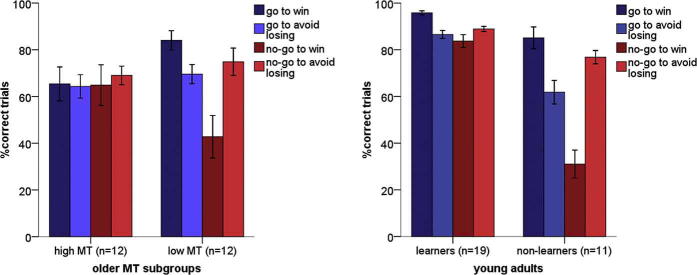


Performance heterogeneity in these younger adults has previously been described, whereby some individuals performed well in all conditions of the task (so-called “learners”; 19 of 30 participants) and others in whom instrumental learning was unsuccessful (so-called “nonlearners”; 11 of 30 participants), where these differences were related to stronger Pavlovian biases in nonlearners (Guitart-Masip et al., 2012b). We found that performance in older adults in the low-MT subgroup resembled that of younger nonlearners whereby Pavlovian response biases dominated performance (Supplementary Fig. 2). In contrast, performance in older adults in the high MT subgroup more closely resembled that of younger adult learners. However, whereas overall performance levels were higher in younger learners compared to nonlearners (89% versus 66% respectively, independent-samples t test t(28) = 10.79, *p* < 0.0005), older adults in the high MT subgroup demonstrated a trade-off between Pavlovian biases (in this case, GW) and instrumental learning (in this case, NGW) such that overall performance levels did not differ between the older groups (66% versus 68% respectively, independent samples t test t(22) = 0.41, *p* = 0.685).

### 3.4 Age differences of SN/VTA structural integrity and relationship with performance

In contrast to the strong relationship between higher NGW performance and higher SN/VTA structural integrity in older adults, no such correlation existed among younger adults (n = 30), nor indeed with any of the task conditions (partial Pearson's correlations with age and SN/VTA volume as covariates: GW, r = −0.12, *p* = 0.543; GAL, r = 0.01, *p* = 0.970; NGW r = −0.04, *p* = 0.859; NGAL, r = 0.07, *p* = 0.743). Thus SN/VTA integrity predicted individual differences in flexible learning among older but not younger adults (Fisher's r-to-z transformation comparing partial correlation strengths of NGW with MT SN/VTA between younger and older adults, with age and SN/VTA volume as covariates: z = −1.93, *p* = 0.05 2-tailed). Also, in contrast to older adults, in younger adults there was no correlation between the main effect of action and SN/VTA integrity (partial Pearson's correlations with age and SN/VTA volume as covariates: r = −0.003, *p* = 0.988) or the action–valence asymmetry and SN/VTA integrity (partial Pearson's correlations with age and SN/VTA volume as covariates: r = −0.09, *p* = 0.636) in younger adults (Fig. 4B).

To examine age-group differences in SN/VTA integrity, we obtained comparable MT imaging (obtained on the same MRI scanner and using the same acquisition and reconstruction protocols) from a separate cohort of 12 young adults. Here we found significantly higher MT values of SN/VTA in younger adults than in older adults, suggesting that older adults had age-related structural decline of the SN/VTA (independent t test, t(52) = 4.13, *p* < 0.0005). Further analysis of the older MT subgroups with younger adults using a 1-way ANOVA with MT values of the right SN/VTA as the dependent variable and age group as the between-subjects factor confirmed a significant between group difference (F_2,33_ = 60.23, *p* < 0.0001). Post-hoc tests among the 3 groups with Bonferroni correction for multiple comparisons showed that there was a significant difference between MT values in the younger group and low MT group in older adults (*p* < 0.0005) but not between the young group and high MT group in older adults (*p* = 0.081) (Fig. 2D). This suggests interindividual variability of MT values of the right SN/VTA across our older cohort.

### 3.5 Instrumental learning and novelty seeking in older adults

**Supplementary Fig. 3:**
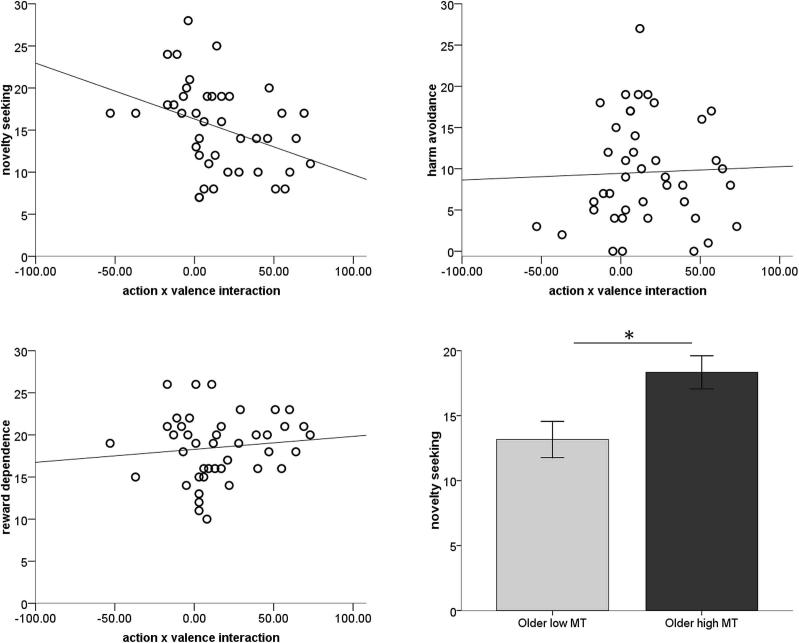


Finally, using a Tridimensional Personality Questionnaire, we assessed the impact of a novelty seeking personality trait on the success of instrumental learning in older age, specifically the ability to overcome the behavioral action–valence interaction. We observed an almost significant trend toward a negative correlation between the behavioral interaction (GW and NGAL > GAL and NGW) and novelty seeking (partial Pearson's correlations controlling for age: r = −0.37, *p* = 0.019), whereas no correlation was observed with the other measured personality traits of harm avoidance (r = 0.008, *p* = 0.959) or reward dependence (r = 0.09, *p* = 0.560) (Supplementary Fig. 3). This suggests that older adults with a more novelty-seeking personality had greater flexible instrumental learning. Interestingly, older participants in the high MT subgroup, that is, participants who showed greater flexibility of instrumental learning, also had higher novelty-seeking scores than older participants in the low-MT group (independent samples t test: t(22) = −2.74, *p* = 0.012) (Supplementary Fig. 3).

## 4 Discussion

Our results reveal that some healthy older adults are unable to flexibly learn 2 responses (go and no-go) for reward within a single task. Through the use of high-resolution quantitative MT imaging, we show that this ability to flexibly learn competing choices for reward is predicted by structural integrity of the SN/VTA and STN. Although we hypothesized that integrity in the SN/VTA would correlate with instrumental learning as demonstrated by our ROI analysis, the additional level of specificity in our whole-brain analysis is remarkable, and suggests that the dopaminergic system may arbitrate between go and no-go choices for reward.

This striking relationship between higher NGW performance and higher SN/VTA integrity was surprising, given previous reports that dopamine promotes “go” and impairs “no-go” learning, for example in patients with Parkinson's disease (Frank et al., 2004). However such studies have tended to explore behavior in 2 conditions, GW and NGAL. Here, using a task that orthogonalizes action (go and no-go) and valence (reward and punishment), we can demonstrate a more precise contribution of the dopaminergic system to this behavioral inflexibility in healthy older individuals (Supplementary Discussion).

It has been suggested that age-related dopamine decline has an impact on the relationship between novelty processing and motivational behavior (Duzel et al., 2010). We found that older participants with less of an asymmetry in action–valence learning had higher novelty-seeking personality scores, and that older adults with higher SN/VTA integrity were more novelty seeking than those with low integrity. These findings may be in keeping with the so-called “exploration bonus” hypothesis that dopamine neurons originating in the SN/VTA can modulate motivational behavior by signaling novel and reward-predicting events (Kakade and Dayan, 2002). It has been reported that novelty-seeking individuals show heightened prediction error signaling in the nucleus accumbens (Abler et al., 2006), as well as increased dopaminergic responses to novelty in the ventral striatum (Zald et al., 2008). Thus, although 1 possible explanation for our findings is that variations in SN/VTA integrity may confer different sensitivities to reward and punishments, the link we identify with novelty seeking could suggest that SN/VTA integrity modulates motivational behavior in this task. Inflexible behavior can arise if participants stick to “go” choices after receiving a reward for a go choice early in the task. In contrast, higher novelty-seeking individuals may be more likely to explore alternative responses (i.e., to sample no-go responses) allowing them to instrumentally learn successfully. However, we acknowledge that a novelty-seeking personality trait is not a direct measurement of exploratory behavior. Alternatively, novelty seeking may be a marker of greater dopaminergic integrity rather than a mechanism related to instrumental learning in the task per se.

Differences in reward sensitivity alone could not fully explain our finding of an interaction between action and valence. If SN/VTA degeneration mainly affected reward sensitivity, then both reward conditions in the task (GW and NGW) would be equally affected, rather than the pattern that we observe of better performance in 1 condition (NGW) at the expense of the other (GW) in individuals with greater SN/VTA integrity. We consider this ability to acquire competing responses for rewards as a marker of flexible learning, although we acknowledge that this does not translate to overall higher performance levels but, rather, to a more even performance across the different contingencies of the task. Future studies relating midbrain structural integrity to other behavioral indices of flexibility, such as reversal learning, could help to further address the nature of this relationship.

In addition to the SN/VTA, the other structure implicated in modulating NGW performance in older adults was the STN. The STN is a biconvex structure that lies superior to the SN/VTA (Dormont et al., 2004). Along with other basal ganglia structures, it too is innervated by dopaminergic fibers from the SN/VTA (Hamani et al., 2004). The STN plays a critical role in action inhibition by relaying a stopping signal (Aron and Poldrack, 2006; Fleming et al., 2010; Frank et al., 2007). This inhibitory network depends on interactions among the STN, inferior frontal gyrus, and supplementary motor area (Aron et al., 2007; Aron and Poldrack, 2006; Coxon et al., 2012; Duann et al., 2009; Jahfari et al., 2011; Swann et al., 2012). Previous work by our own group using the same go/no-go task has shown that inferior frontal gyrus activity is associated with no-go learning and successful instrumental control (Guitart-Masip et al., 2012b). The current structural SN/VTA and STN findings are therefore compatible with a literature relating functional activity in the post-synaptic targets of midbrain nuclei and their related circuits to both response inhibition and instrumental learning. It is also notable that our VBQ analysis localized NGW learning to structural integrity of the right SN/VTA and STN, since inhibitory processing has been reported to evoke a right-lateralized network (Aron et al., 2003; Coxon et al., 2012; Garavan et al., 1999; Zheng et al., 2008).

An important consideration in this study was behavioral and structural differences between younger and older adults. Although overall patterns of performance were similar in younger and older adults (as shown in Fig. 2A and B), some differences emerged, which we speculate are linked to age-related neural differences. At a group level, older adults with the lowest MT values of SN/VTA displayed a behavioral inflexibility, particularly for rewards. These same adults had significantly lower MT values of SN/VTA than younger adults, which might mean they had age-related degeneration of the SN/VTA. Although significantly lower overall performance was observed in the older group, performance in these older individuals was markedly similar to that of younger adults who were unable to learn this task in terms of the observed action–valence interaction during learning. In contrast, performance in older adults with midbrain integrity similar to that of younger adults resembled performance seen in younger adult “learners.” However, it was notable that these older adults who learned to overcome pre-potent response biases did so at the cost of overall task performance. This trade-off between instrumental and Pavlovian systems was not evident in younger adults who instrumentally learned successfully. One possible explanation for this is the involvement of other brain regions in younger adults performing this task. For example, it has been shown that young adults who are able to instrumentally learn in this task show heightened activity in the inferior frontal gyrus (Guitart-Masip et al., 2012b). Future studies designed to directly test age differences in this structure–function relationship could elucidate this further.

One advantage of our study was the use of high-quality MT images to accurately identify the SN/VTA and R2* images to define the STN. The MT contrast is particularly suited to visualizing brainstem structures as it provides better gray/white matter contrast than the standard T1w MRI contrast (Helms et al., 2009). MT measures macromolecule concentration and thus reflects the properties of bound protons in structures such as myelin (Tofts, 2003), axons (Klistorner et al., 2011), cell membrane proteins, and phospholipids (Bruno et al., 2004; Wolff and Balaban, 1989). Moreover, reduced MT in the SN/VTA has been described in Parkinson's disease and is proposed to reflect the loss of dopamine neurons (Eckert et al., 2004) (Tambasco et al., 2011). We found that some, but not all, older adults had lower structural integrity of the SN/VTA than did younger adults. This suggests interindividual variability of SN/VTA structural integrity among older adults, and possibly relates to variable dopamine decline as a function of age, although we acknowledge that the exact pathology underlying alterations in the MT signal in normal aging remains unknown. Future studies combining MT imaging with other imaging modalities (e.g., positron emission tomography) and histological evidence will help to provide greater insight into the interpretation of MT values of dopaminergic brainstem structures.

In summary, the new perspective highlighted here is that individual differences of SN/VTA integrity contribute to learning flexibility by allowing older individuals to overcome response biases. In contrast, structural integrity of SN/VTA did not predict instrumental learning in younger adults, suggesting that instrumental learning in older age is sensitive to structural changes in the dopaminergic midbrain.

## Disclosure statement

All authors report no actual or potential conflicts of interest.

## Figures and Tables

**Table 1:** Multiple regression results for each predictor variable for no-go to win performance

Model	Predictor variable	β	*p*
Model 1	T1	−0.07	0.66
	R2*	0.08	0.59
	Age	0.20	0.18
	Vol	0.26	0.10
	MT	0.38	0.02
Model 2	R2*	0.08	0.57
	Age	0.20	0.19
	Vol	0.24	0.10
	MT	0.41	0.07
Model 3	Age	0.22	0.12
	Vol	0.24	0.10
	MT	0.40	0.007
Model 4	Vol	0.21	0.17
	MT	0.40	0.01
Model 5	MT	0.46	0.002

The MT value of the SN/VTA was the only significant contributing variable to no-go to win performance in each model. Key: MT, magnetization transfer; SN/VTA, substantia nigra/ventral tegmental area; Vol, SN/VTA volume; T1, T1 quantitative MRI map; R2∗, R2∗quantitative MRI map.

**Table 2:** Voxel-based quantification results for no-go to win positive correlation with gray matter MT images

Region	No. of voxels	MNI co-ordinates			t	z
		x (mm)	y (mm)	z (mm)		
Right SN/VTA and STN	33	9	−16.5	−7.5	3.73	3.39
left cerebellum	16	−37.5	−64.5	−39	3.57	3.27
left putamen	13	−27	3	−4.5	3.51	3.22

Peak level results are shown for all clusters of more than 10 voxels; *p* < 0.001 uncorrected at the whole-brain level. Key: MNI, Montreal Neurological Institute; MT, magnetization transfer; SN/VTA, substantia nigra/ventral tegmental area; STN, subthalamic nucleus.
