# Supplementary material for: Structural integrity of the substantia nigra and subthalamic nucleus predicts flexibility of instrumental learning in older-age individuals
Source: Neurobiol Aging. 2013 Oct;34(10):2261–70. doi: 10.1016/j.neurobiolaging.2013.03.030 (PMC3713434; doi:10.1016/j.neurobiolaging.2013.03.030)
Supplement: Supplementary Data [file mmc1.docx]

**Structural integrity of the substantia nigra and subthalamic nucleus predicts the flexibility of instrumental learning in older age**

Rumana Chowdhury*^1,2^, Marc Guitart-Masip^2^, Christian Lambert^2^, Raymond J Dolan^2^, Emrah Düzel^1,3,4^

^1^ Institute of Cognitive Neuroscience, University College London, 17 Queen Square, London, UK

^2^ Wellcome Trust Centre for Neuroimaging, Institute of Neurology, University College London, 12 Queen Square, London, UK

^3^ Otto-von-Guericke-University Magdeburg, Institute of Cognitive Neurology and Dementia Research, Leipziger Str. 44, 39120 Magdeburg, Germany

^4^ German Center for Neurodegenerative Diseases (DZNE), Magdeburg, Germany

*Dr Rumana Chowdhury (corresponding author)

Institute of Cognitive Neuroscience

17 Queen Square

London WC1N 3AR

Email: [rumana.neuro@gmail.com](mailto:rumana.neuro@gmail.com)

Tel: +44 (0)20 7679 4727

**Abstract**

Flexible instrumental learning is required to harness the appropriate behaviours to obtain rewards and to avoid punishments. The precise contribution of dopaminergic midbrain regions (substantia nigra / ventral tegmental area, SN/VTA) to this form of behavioural adaptation remains unclear. Normal aging is associated with a variable loss of dopamine neurons in the SN/VTA. We therefore tested the relationship between flexible instrumental learning and midbrain structural integrity. We compared task performance on a probabilistic monetary go/no-go task involving trial and error learning of: ‘go to win’, ‘no-go to win’, ‘go to avoid losing’ and ‘no-go to avoid losing’ in 42 healthy older adults with previous behavioural data from 47 young adults. Quantitative structural magnetization transfer images were obtained to index regional structural integrity. On average, both some young and older participants demonstrated a behavioural asymmetry whereby they were better at learning to act for reward (‘go to win’ > ‘no-go to win’), but better at learning not to act to avoid punishment (‘no-go to avoid losing’ > ‘go to avoid losing’). Older but not young participants with greater structural integrity of the SN/VTA and the adjacent subthalamic nucleus could overcome this asymmetry. We show that inter-individual variability amongst healthy older adults of the structural integrity within the SN/VTA and subthalamic nucleus relates to effective acquisition of competing instrumental responses.

**Keywords:** aging; instrumental learning; magnetization transfer; novelty seeking; substantia nigra

**1. Introduction**

To efficiently harvest reward and avoid punishment, humans need to learn appropriate instrumental responses (Dickinson A, & Balleine, B, 2002) (O'Doherty J et al., 2004). Recent data suggest that this basic form of behavioural adaption is surprisingly inflexible in humans (Guitart-Masip M, QJM Huys et al., 2012). While healthy young human adults readily learn to act in order to obtain a reward or not to act in order to avoid a punishment, they have difficulties learning to act in order to avoid a punishment and not to act to obtain a reward (Guitart-Masip M, QJM Huys *et al.*, 2012). This inflexibility in learning suggests that signals that predict rewards are prepotently associated with behavioural activation promoting approach behaviour whereas signals associated with punishments are intrinsically coupled to behavioural inhibition promoting avoidance. These behavioural tendencies can be described as Pavlovian biases that corrupt the flexibility of instrumental learning (Dayan P et al., 2006; Gray JA, and McNaughton, M., 2000). Computational modelling in young adults has shown that the observed pattern of behaviour is captured by a model incorporating a Pavlovian bias, where the strength of this bias is related to failure to learn the conflicting conditions: no-go to win and go to avoid losing (Guitart-Masip M, QJM Huys *et al.*, 2012).

The substantia nigra/ventral tegmental area (SN/VTA) of the midbrain, the origin of dopaminergic projections, is important for instrumental learning (Salamone JD et al., 2005; Schultz W et al., 1997) including signalling reward predictions errors (Schultz W *et al.*, 1997), energizing actions (Niv Y et al., 2007) and driving novelty-related exploratory behaviour (Duzel E et al., 2010; Lisman J et al., 2011). In humans, dopaminergic medication after learning influences the brain responses to action and reward anticipation (Guitart-Masip M, R Chowdhury et al., 2012). Importantly, the SN/VTA undergoes degeneration with aging (Bäckman L et al., 2006; Fearnley JM and AJ Lees, 1991; Vaillancourt DE et al., 2012). Age-differences in instrumental learning have been linked to functional activity in dopaminergic target regions including the striatum and prefrontal cortex (Samanez-Larkin GR et al., 2010) (Mell T et al., 2009) (Fera F et al., 2005) (Aizenstein HJ et al., 2006). Structural degeneration of the SN/VTA and associated circuits can be indexed *in vivo* by magnetization transfer (MT) imaging, where lower MT values reflect decreased structural integrity (Düzel S et al., 2008; Eckert T et al., 2004; Tambasco N et al., 2011).

The goal of this study was to relate individual differences of SN/VTA integrity in older age to flexible instrumental learning for competing responses (“to act” or “not to act”) to rewards and punishments. Furthermore, to explore age-group comparisons of learning and structural integrity of SN/VTA, we obtained separate data from young adults. We hypothesized that older adults with higher SN/VTA integrity would show greater learning flexibility. Thus instrumentally learning to act in order to avoid a punishment and not to act to obtain a reward would be equivalent to learning to act in order to obtain a reward or not to act in order to avoid a punishment, the latter being Pavlovian response biases that tend to dominate learning. We also obtained trait measures of novelty seeking in older adults to test the relationship with instrumental learning and structural integrity.

**2. Materials and Methods**

**2.1 Participants**

**Older participants:** 42 healthy older adults aged 64-75 years (mean 69.12 yrs SD 3.44; 29 females; 40 right-handed) were recruited via our departmental website, advertisement in local public buildings and by word of mouth. Individuals were initially screened by telephone and excluded if they had any of the following: current or past history of neurological, psychiatric conditions or endocrinological disorders, metallic implants, tinnitus, major visual impairment, history of drug addiction. To control for vascular risk factors, individuals known to have had a stroke or transient ischemic attack, myocardial infarction or other significant cardiovascular history, diabetes mellitus or hypertension requiring more than one anti-hypertensive medication were not eligible for participation. All participants undertook a neuropsychological test battery to ensure intact global cognitive performance (**Supplementary Table 1**). On the basis of this no participants were excluded from the analysis (all participants scored within 1.5 SDs of the age-related norm for each test). All participants had a normal neurological examination (performed by a physician R.C.) ensuring participants did not have concurrent undiagnosed neurological conditions. MRI scans were visually inspected to ensure no participants had severe white matter changes or other major lesions. Clinical examination, neuropsychological testing, the go/no-go task and structural MRI scanning were all performed in a single four hour session. Written informed consent was obtained from all participants. The study received ethical approval from the North West London Research Ethics Committee 2.

**Young participants:** Data from two previously published experiments performed at the host institution were obtained to enable separate age-comparisons of behavioural data and MRI data. In one, behavioural data from 47 healthy young adults (28 female; mean age 23.1 years, SD 4.1) performing the same go/no-go task was obtained allowing comparisons of behavioural performance between young and older adults (Guitart-Masip M, QJM Huys *et al.*, 2012). Structural neuroimaging including MT imaging was available for 30 of these young adults, which we used to examine the correlation between SN/VTA integrity and task performance in young adults. These scans were obtained on a different MRI scanner (3-T Siemens Allegra) using a different acquisition protocol that did not include B1 correction (see (Guitart-Masip M, QJM Huys *et al.*, 2012) for details), thus direct age-comparisons of actual MT values could not be made with this dataset and ours.

Therefore in the second study, neuroimaging data from 12 healthy young adults (6 females; mean age 33.8, SD 12.84) using the same MRI scanner and imaging sequence was obtained to allow comparison of MT values of SN/VTA between young and older adults (Lambert C et al., 2012).

**2.2 Go/no-go task**

Participants performed a probabilistic monetary go/no-go task as described in Guitart-Masip et al., (2012) (Guitart-Masip M, QJM Huys *et al.*, 2012) (**Fig. 1**). The correct response (to execute or withhold an action) to four cues (abstract fractal images) had to be learnt through trial and error, in order to win or avoid losing money. Participants were told that at the start of the task they would not know the correct responses (to press or not press a button) for each image but that these would become clear through trial and error. After seeing an image (1000ms), there was a variable interval (250-2000 ms) after which participants were presented with a circle (target detection, 1500ms), at which point they had to either press a button (go) with their dominant hand to indicate the target side within 1000ms or not press a button (no-go) . Following this, the outcome was depicted for 1000ms by a green up-pointing arrow (indicating a win of £1), a red down-pointing arrow (indicating a loss of £1) or a yellow horizontal bar (neither win nor lose). The outcome was probabilistic, whereby in the win conditions, 80% of correct choices and 20% of incorrect choices were rewarded (the remaining 20% of correct and 80% of incorrect choices leading to a neutral outcome). In the lose conditions, 80% of correct choices and 20% of incorrect choices avoided punishment (the remaining 20% of correct and 80% of incorrect choices leading to a neutral outcome). The probabilistic nature of the task was made clear to participants in the written and verbal instructions prior to the task. Thus, the task consisted of four trial types depending on the nature of the fractal cue presented at the beginning of the trials:

- Press the correct button in the target detection task to gain a reward (go to win, GW)
- Press the correct button in the target detection task to avoid punishment (go to avoid losing, GAL)
- Do not press a button in the target detection task to gain a reward (no-go to win, NGW)
- Do not press a button in the target detection task to avoid punishment (no-go to avoid losing, NGAL)

The task consisted of 240 trials (60 trials for each of the four conditions, presented in a randomised fashion) and lasted approximately 35 minutes. At the beginning of the task, participants were told they could win between £5 to £15 and were given their earnings on task completion. Prior to the actual task, participants undertook a brief training session of ten practice trials in which only the target detection circles were presented. Participants were instructed to press the corresponding button for every target (left arrow key on the keyboard if the target appeared on the left of the screen and visa versa for right). This allowed participants to familiarise themselves with the appropriate buttons on the computer keyboard and obtain an overall feel for the speed of the task without exposure to any of the cues used in the main task.

**2.3 Tridimensional Personality Questionnaire**

Each participant completed the Tridimensional Personality Questionnaire (TPQ) (Cloninger CR, 1987). This self-report questionnaire consists of 100 true-false items measuring three personality traits: novelty-seeking, harm-avoidance and reward-dependence.

**2.4 MRI scanning**

A high-resolution structural MRI dataset for each participant was obtained on a 3.0T MRI scanner (Magnetom TIM Trio, Siemens Healthcare, Erlangen, Germany) using a 32-channel head coil. A structural multi-parameter map protocol employing a 3D multi-echo fast low angle shot (FLASH) sequence at 1mm isotropic resolution was used to acquire MT weighted (echo time, TE, 2.2-14.70ms, repetition time, TR, 23.7ms, flip angle, FA, 6 degrees), proton density weighted (TE 2.2-19.7ms, TR 23.7ms, FA 6 degrees) and T1 weighted (TE 2.2-14.7ms, TR 18.7ms, FA 20 degrees) images (Helms G et al., 2008). B1 mapping (TE 37.06 and 55.59ms, TR 500ms, FA 230:-10:130 degrees, 4mm^3^ isotropic resolution) was acquired to correct the T1 maps for inhomogeneities in the transmit radiofrequency field (Lutti A et al., 2010). A double-echo FLASH sequence (TE1 10ms, TE2 12.46ms, 3 x 3 x 2 mm resolution and 1mm gap) was used to measure local field inhomogeneities and correct for the image distortions in the B1 mapping data.

Using in-house code, the MT, T1 and R2* (1/T2*) quantitative maps were extracted for each subject from the anatomical scans described above. Proton density scans were not used for any analyses but were acquired as they are crucial for estimating MT and T1 parameters (for full details regarding the generation of quantitative maps see (Helms G *et al.*, 2008)). MT, T1 and R2* values reflect structural integrity (Düzel S *et al.*, 2008; Eckert T *et al.*, 2004; Tambasco N *et al.*, 2011; Wolff SD and RS Balaban, 1989), myelin and iron content respectively (Draganski B et al., 2011; Martin WRW, 2009; Martin WRW et al., 2008).

**2.5 Imaging analysis**

Data processing and analysis was performed using Statistical Parametric Mapping software (SPM8; Wellcome Trust Centre for Neuroimaging, London, UK) and MATLAB 7.8 (Mathworks, Sherborn, MA, USA). Two independent analyses were conducted with the structural MRI data from older adults. The first was a region-of-interest analysis (ROI) of the SN/VTA. The second was a whole-brain voxel-based analysis.

**2.6 Definition of regions of interest**

**Substantia nigra/ventral tegmental area (SN/VTA):** The medial and lateral boundaries of the SN/VTA were defined on each participants’ MT-weighted image where it is easily distinguishable from the surrounding tissues due to its bright grey colour in contrast to the adjacent cerebral peduncle. For each subject, this region was manually defined on every visible slice, usually between seven to ten slices as per Düzel et al (Düzel S *et al.*, 2008) using MRIcro (Rorden C BM, 2000). A single slice example from a single subject is shown in **Fig. 3A** (**Supplementary Fig. 1** is an example of all slices from a single subject). For each subject, their ROI was projected as an overlay on their MT, T1 and R2* maps to obtain a mean value for the region. Bilateral SN/VTA values in older adults, calculated by averaging right and left SN/VTA values, were as follows: MT mean 0.93 (SD 0.070), T1 mean 1129.31 (SD 51.23), R2* mean 0.028 (SD 0.0048).

**Subthalamic nucleus (STN):** The STN was manually segmented for each subject using the software package ITK-SNAP (Yushkevich PA et al., 2006) as described in Lambert et al (2012) (Lambert C *et al.*, 2012). Briefly, using R2* maps, it appears as a hyperintense region. The borders of the STN were defined as the zona incerta superiorly and immediately medially; preleminiscal radiations, posterior-lateral hypothalamus and red nucleus further medially and cerebral peduncle laterally. The inferior tip lies on the superior aspect of the substantia nigra at the level of the optic tract. See **Supplementary Fig. 1** for a single subject example.

Ten randomly selected SN/VTA and STN ROIs were segmented by a second trained individual (authors’ C.L. and R.C respectively), showing high inter-rater reliability (SN/VTA: Intraclass correlation = 0.87, p < 0.0005; STN: Intraclass correlation = .98, p < 0.0005).

**2.7 Magnetization transfer (MT) subgroups**

We obtained MT data for 12 young adults (mean age 33.8 years SD 12.84, 6 females) from a separate published experiment (Lambert C *et al.*, 2012). For comparison we formed two subgroups each consisting of 12 older adults matched for age and gender (10 females per group), that differed significantly in MT values of the right SN/VTA (independent samples t-test, 2-tailed: t(22) = -9.93, p < 0.0001). For these subgroups, 12 older adults with the highest and lowest MT values of the right SN/VTA were selected to form a ‘high MT’ group (MT (mean, SD): 0.98, 0.038; age (mean, SD): 69.33 years, 2.74) and ‘low MT’ group respectively (MT (mean, SD): 0.84, 0.023; age (mean, SD): 70.08 years, 3.34). We used right SN/VTA for post hoc tests of the MT subgroups based on the major VBQ finding of a correlation between right SN/VTA integrity and NGW performance. We used these subgroups for three analyses: first, to further explore the relationship between MT and behaviour within older adults only; second, to compare MT values of the SN/VTA between young and older adults; and third to compare novelty seeking scores within older adults only.

**2.8 Voxel based quantification**

To explore the regional specificity of the correlation between SN/VTA integrity and task performance in older adults, a method recently termed Voxel Based Quantification (VBQ) was used (Draganski B *et al.*, 2011). This allows whole brain statistical analysis of quantitative MRI parameters such as MT. The methodology was adapted from Draganski *et al.,* (2011) with a few adjustments specific to the current cohort summarised as follows. In brief, unified segmentation was used to classify MT maps into grey matter, white matter and cerebrospinal fluid (Ashburner J and KJ Friston, 2005). Whilst better segmentation of subcortical regions can be attained using MT rather than T1 maps (Helms G et al., 2009), visual inspection revealed that the SN/VTA region was often incomplete and misclassified as white matter. Therefore, in subject space the manually defined SN/VTA ROI was added to each un-modulated grey matter mask and subtracted from the white matter. These maps were adjusted to ensure that all voxels remained in the range from zero to one. Using a diffeomorphic registration algorithm (DARTEL) the MT white and gray matter maps were warped to a common template (Ashburner J, 2007). Modulation was achieved by multiplying these warped images with their Jacobian determinants. Finally, weighted average MT maps were created as previously described (Draganski B *et al.*, 2011) and smoothed with an isotropic Gaussian kernel of 6mm full width at half maximum.

**2.9 Statistical analysis**

Performance in each of the four task conditions was calculated as the percentage of correct responses and analysed using a repeated measures ANOVA with action (go/no-go) and valence (win/avoid loss) as the within-subjects factors. To compare performance between older MT subgroups, MT-group (low/high) was added as a between-subjects factor. To compare performance between all young and all older adults, age-group (young/older) was added as a between-subjects factor. To further explore behavioural response biases in go/no-go task performance in older age, we calculated the following measures using the total number of correct trials per condition: main effect of action (GW+GAL-NGW-NGAL), main effect of valence (GW+NGW-GAL-NGAL) and an interaction between action and valence (GW+NGAL-NGW-GAL). Partial Pearson’s correlations (controlling for age and SN/VTA volume) were used to correlate response biases with SN/VTA MT values (significance level set at p < 0.017 after Bonferroni correction for three tests) and to assess the relationship between the behavioural interaction and personality measures of novelty seeking, reward dependence and harm avoidance (significance level set at p < 0.017 after Bonferroni correction for three tests). All reported significance values are two-tailed.

For structural imaging parameters of SN/VTA, linear multiple regression analyses were performed using Statistical Package for the Social Sciences (SPSS, Version 17.0). We used a backwards model to conduct a separate analysis for each of the four task conditions (GW, GAL, NGW, NGAL) where performance (percentage of total correct responses) in these conditions was used as the dependent variable. The five independent variables in each model were the three imaging parameter values of bilateral SN/VTA (MT, T1 and R2* values), volume of the SN/VTA and age. The significance level for each model was set at p < 0.0125 (Bonferroni correction for four models). To address co-variance between MT and T1 values (**Supplementary Table 2**), we also report separate correlations between neuroimaging parameters and task performance (**Supplementary Table 3**). All reported significance values are two-tailed.

The VBQ analysis was only performed for the significant task condition (no-go to win) and image type (MT) from the behavioural regression analyses to minimise the number of voxel-based analyses. The calculated weighted average MT maps were analysed in a multiple regression model in SPM8. A single analysis was performed using a design matrix containing performance in all four task conditions (GW, GAL, NGW, NGAL) as separate covariates and age, gender and total intracranial volume (sum of grey matter, white matter and CSF) as regressors of no interest. We included performance in all four task conditions in a single model as a more stringent test to identify the unique variance associated with NGW performance over and above performance in the other conditions (**Supplementary Table 4** shows no sigfificant covariance between these measures). An explicit mask created from the grey matter probability maps thresholded at 0.2 was applied. Uncorrected whole brain p-values <0.001 for clusters greater than 10 voxels are reported. We created SN/VTA and STN masks for small volume correction using individual participants’ manually defined ROI’s, normalised to MNI space using DARTEL and group-averaged. A statistical threshold of p < 0.05 after family-wise error correction was used for the hypothesis-based small volume correction analyses.

**3. Results**

**3.1 Go/no-go task performance in older adults**

Older participants were, on average, more accurate at go choices when the outcome was a reward (GW) and at no-go choices when the outcome was avoidance of losses (NGAL) (two (go/no-go) by two (win/avoid loss) repeated measures ANOVA: action by valence interaction: F(1,41) = 12.55, p=.001; GW versus GAL: t(41) = 2.26, p=.029; NGW versus NGAL: t(41) = -3.20, p=.003; **Fig. 2A**). We also found a main effect of action indicating participants were better at learning go compared to no-go choices (F(1,41) = 7.29, p=.01). There was no main effect of valence (F(1,41) = 1.87, p=.18). These results demonstrate that older adults had a marked asymmetry in their learning behaviour.

Older adults showed a preponderant initial bias towards go responses (**Fig. 2A)** (one sample t-test for performance in the first 10 trials: GW t(41) = 6.578, p = .000; GAL t(41) = 2.249, p = .030). In contrast, performance in the first ten trials was at chance for the NGAL condition (t(41) = 0.638, p = .527) and significantly below chance for NGW (t(41) = -4.365, p = .000). This suggests a persisting action bias in the reward condition, whereas with loss a bias towards no-go responses emerged during learning. Over the course of the task, learning occurred in all conditions (**Supplementary Table 5**).

**3.2 Structural neuroimaging in older adults**

**Region-of-interest analysis:** For each experimental condition amongst older adults, we constructed a multiple regression model with task performance as the dependent variable and SN/VTA imaging parameter values (MT, T1, R2*), age and SN/VTA volume as independent variables. These models only explained variance in NGW performance where the best model contained MT as the only explanatory variable (standardised Beta MT = 0.46, p = .002, R square = 0.21). The additional variables did not add explanatory power (**Table 1**). Thus, higher SN/VTA integrity predicted an ability to learn to inhibit an action to obtain reward. **Fig. 3A** shows this correlation, which remained significant after controlling for both total intracranial volume and size of the SN/VTA (partial Pearson’s r = 0.39, p = .014). Regression models for the remaining task conditions were not significant suggesting that neither structural integrity, iron or myelin content of SN/VTA were associated with learning the GW, GAL or NGAL conditions (**Supplementary Table 6**).

We next analysed how SN/VTA integrity related to the ability to overcome response biases. The action bias (go > no-go performance for both wins and losses) was negatively correlated with SN/VTA integrity (r = -0.45, p = 0.003, **Fig. 4A;** partial correlation controlling for age and SN/VTA volume: r = -0.40, p = .011) suggesting that only those individuals with high SN/VTA integrity were able to overcome this action bias. Moreover, the negative correlation between the interaction in task performance (go to win and no-go to avoid losing > no-go to win and go to avoid losing performance) and SN/VTA integrity suggests the action-valence learning asymmetry could also be overcome with higher SN/VTA integrity (r = -0.42, p = 0.006, **Fig. 4A;** partial correlation controlling for age and SN/VTA volume: r =-0.35, p = .028). There was no correlation between SN/TA integrity and the main effect of valence (r = 0.22, p = .155; partial correlation controlling for age and SN/VTA volume: r = 0.27, p = .091). We found no evidence that working memory capacity contributed to the relationship between SN/VTA integrity and the pattern of task performance amongst older adults (**Supplementary Results**).

These results were also reflected in the older adult MT subgroup analyses, whereby we formed two gender-matched groups of older adults with the highest and lowest MT values of SN/VTA. We performed a repeated measures ANOVA as before with action (go/ no-go) and valence (win/ avoid loss) as within-subjects factors but additionally included MT group (low/ high) as a between-subjects factor. **Fig. 2C** shows the striking behavioural asymmetry between action and valence learning was present in the low MT group but not in the high MT group (3-way action by valence by MT-group interaction: F(1,22) = 5.25, p = .032). Older individuals with low SN/VTA integrity were inflexible in learning the reward conditions: they readily learned the GW condition but were less able to concurrently learn the NGW condition. In contrast, older individuals with high SN/TA integrity were instrumentally more flexible, i.e. acquired both go and no-go responses concurrently to obtain rewards. However, higher flexibility in the high MT group came at a cost for GW performance (trend towards a negative correlation between GW and NGW performance (r = -0.28, p =0.071) but not between GAL and NGAL (r =0.13, p = 0.42). This suggests a trade-off between the ability to learn competing responses in the reward conditions. Similar to the assessment of behaviour across all 42 older adults, this analysis of the MT-subgroups also demonstrated a trend towards a main effect of action (F(1,22) = 3.16, p = .089), a significant action by valence interaction (F(1,22) = 8.28, p = .009) and no main effect of valence (F(1,22) = 1.47, p = .24). Overall, these results suggest that amongst older individuals, those with higher integrity of the SN/VTA were able to overcome their initial response biases leading to more flexible instrumental learning, evidenced by a more even performance across the different action-valence contingencies. Since the behavioural interaction was mostly driven by GW and NGW learning, these correlations also show that higher SN/VTA integrity confers flexibility by an improvement in NGW learning but with a concurrent slight decline in GW learning.

**Voxel-based quantification:** To address potential bias from a ROI-analysis, and assess the anatomical specificity in the relationship between NGW performance and SN/VTA, we used a whole brain voxel-based quantification (VBQ) analysis. This showed that positive correlations between NGW performance and MT values were restricted to a region that included the right SN/VTA and STN (**Fig. 3B**), with smaller clusters in the left cerebellum and left putamen only (**Table 2**).

For the SN/VTA and STN cluster, we quantified the percentage of overlap with probability maps of each anatomical region and found that 17.4% of the cluster overlapped with the STN, compared to 47.6% overlap with the SN/VTA. Using these probability maps, the multiple regression VBQ analysis of NGW performance and MT values of the right SN/VTA survived a hypothesis-based small volume correction (p <0.05, FWE-corrected, Z_max_ = 3.39, x = 9, y = -17, z = -8). The same was true for the right STN (p <0.05, FWE-corrected, Z_max_ = 3.33, x = 11, y = -17, z = -8).

**3.3 Comparison of task performance between young and older adults**

To directly compare task performance between age-groups, we obtained data from a separate experiment in which the same behavioural task was performed by 47 young adults, of which 30 underwent MT imaging (a detailed description of behaviour amongst these young adults can be found in (Guitart-Masip M, QJM Huys *et al.*, 2012)). A two by two repeated measures ANOVA with action (go/no-go) and valence (win/avoid loss) as within subjects-factors, and age group (young/older) as between-subjects factor showed a main effect of action (F (1,87) = 21.75, p = .000), main effect of valence (F(1.87)=4.17, p = .044) and significant action by valence interaction (F(1,87) = 47.23, p = .000) but no significant interaction of any factors with age group. Thus the overall pattern of performance showing a marked behavioural asymmetry was present in both young and older adults (**Fig. 2B)**. Performance averaged over all task conditions was worse in older adults (main effect of age F(1,88) = 15.15, p <0.0005).

Performance heterogeneity in these young adults has previously been described, where some individuals performed well in all conditions of the task (so-called ‘learners’, 19/30 participants) and others in whom instrumental learning was unsuccessful (so-called ‘non-learners’, 11/30 participants), where these differences were related to stronger Pavlovian biases in non-learners. (see (Guitart-Masip M, QJM Huys *et al.*, 2012)). We found that performance in older adults in the low MT subgroup resembled that of young non-learners whereby Pavlovian response biases dominated performance (**Supplementary Fig. 2**). In contrast, performance in older adults in the high MT subgroup more closely resembled that of young adult learners. However, whilst overall performance levels were higher in young learners compared to non-learners (89% vs. 66% respectively, independent samples t-test t(28) = 10.79, p <0.0005), older adults in the high MT subgroup demonstrated a trade-off between Pavlovian biases (in this case, GW) and instrumental learning (in this case, NGW) such that overall performance levels did not differ between the older groups (66% vs. 68% respectively, independent samples t-test t(22) = 0.41, p = .685).

**3.4 Age differences of SN/VTA structural integrity and relationship with performance**

In contrast to the strong relationship between higher NGW performance and higher SN/VTA structural integrity in older adults, no such correlation existed amongst young adults (n = 30), nor indeed with any of the task conditions (partial Pearson’s correlations with age and SN/VTA volume as covariates: GW, r = -0.12, p = .543; GAL, r = 0.01, p =.970; NGW r = -0.04, p = .859; NGAL, r = 0.07, p = .743). Thus SN/VTA integrity predicted individual differences in flexible learning amongst older but not young adults (Fisher’s r-to-z transformation comparing partial correlation strengths of NGW with MT SN/VTA between young and older adults, with age and SN/VTA volume as covariates: z = -1.93, p = .05 two-tailed). Also in contrast to older adults, in young adults there was no correlation between the main effect of action and SN/VTA integrity (partial Pearson’s correlations with age and SN/VTA volume as covariates: r = -0.003, p = .988) or the action by valence asymmetry and SN/VTA integrity (partial Pearson’s correlations with age and SN/VTA volume as covariates: r = -0.09, p = .636) in young adults (**Fig. 4B**).

To examine age-group differences in SN/VTA integrity, we obtained comparable MT imaging (obtained on the same MRI scanner and using the same acquisition and reconstruction protocols) from a separate cohort of 12 young adults. Here we found significantly higher MT values of SN/VTA in young adults than in older adults, suggesting that older adults had age-related structural decline of the SN/VTA (independent t-test, t(52) = 4.13, p < 0.0005). Further analysis of the older MT subgroups with young adults using a one-way ANOVA with MT values of the right SN/VTA as the dependent variable and age-group as the between-subjects factor confirmed a significant between group difference (F(2,33) = 60.23, p < 0.0001). Post hoc tests between the three groups with Bonferroni correction for multiple comparisons showed that there was a significant difference between MT values in the young group and low MT group in older adults (p < 0.0005) but not between the young group and high MT group in older adults (p = .081) (**Fig. 2D**). This suggests inter-individual variability of MT values of the right SN/VTA across our older cohort.

**3.5 Instrumental learning and novelty seeking in older adults**

Finally, using a Tridimensional Personality Questionnaire we assessed the impact of a novelty seeking personality trait on the success of instrumental learning in older age, specifically the ability to overcome the behavioural action-valence interaction. We observed an almost significant trend towards a negative correlation between the behavioural interaction (GW and NGAL > GAL and NGW) and novelty seeking (partial Pearson’s correlations controlling for age: r = -0.37, p = .019) whereas no correlation was observed with the other measured personality traits of harm avoidance (r = 0.008, p = .959) or reward dependence (r = 0.09, p = .560) **(Supplementary Fig. 3)**. This suggests that older adults with a more novelty seeking personality had greater flexible instrumental learning. Interestingly, older participants in the high MT subgroup, that is participants who showed greater flexibility of instrumental learning, also had higher novelty seeking scores than older participants in the low MT group (independent samples t-test: t(22) = -2.74, p = .012) **Supplementary Fig. 3)**.

**4. Discussion**

Our results reveal that some healthy older adults are unable to flexibly learn two responses (go and no-go) for reward within a single task. Through the use of high resolution quantitative MT imaging we show that this ability to flexibly learn competing choices for reward is predicted by structural integrity of the SN/VTA and STN. Although we hypothesized that integrity in the SN/VTA would correlate with instrumental learning as demonstrated by our ROI analysis, the additional level of specificity in our whole-brain analysis is remarkable and suggests that the dopaminergic system may arbitrate between go and no-go choices for reward.

This striking relationship between higher NGW performance and higher SN/VTA integrity was surprising given previous reports that dopamine promotes ‘go’ and impairs ‘no-go’ learning, for example in patients with Parkinson’s disease (Frank MJ et al., 2004). However such studies have tended to explore behavior in two conditions, GW and NGAL. Here, using a task which orthogonalises action (go and no-go) and valence (reward and punishment), we can demonstrate a more precise contribution of the dopaminergic system to this behavioural inflexibility in healthy older individuals (**Supplementary Discussion**).

It has been suggested that age-related dopamine decline has an impact on the relationship between novelty processing and motivational behavior (Duzel E *et al.*, 2010). We found that older participants with less of an asymmetry in action-valence learning had higher novelty seeking personality scores and older adults with higher SN/VTA integrity were more novelty seeking than those with low integrity. These findings may be in keeping with the so-called ‘exploration bonus’ hypothesis that dopamine neurons originating in the SN/VTA can modulate motivational behaviour by signalling novel and reward-predicting events (Kakade S and P Dayan, 2002). It has been reported that novelty seeking individuals show heightened prediction error signalling in the nucleus accumbens (Abler B et al., 2006), as well as increased dopaminergic responses to novelty in the ventral striatum (Zald DH et al., 2008). Thus whilst one possible explanation for our findings is that variations in SN/VTA integrity may confer different sensitivities to reward and punishments, the link we identify with novelty seeking could suggest that SN/VTA integrity modulates motivational behaviour in this task. Inflexible behaviour can arise if participants stick to go choices after receiving a reward for a go choice early in the task. In contrast, higher novelty seeking individuals may be more likely to explore alternative responses (i.e. sample no-go responses) allowing them to successfully instrumentally learn. However, we acknowledge that a novelty seeking personality trait is not a direct measurement of exploratory behaviour. Alternatively, novelty seeking may be a marker of greater dopaminergic integrity rather than a mechanism related to instrumental learning in the task per se.

Differences in reward sensitivity alone could not fully explain our finding of an interaction between action and valence. If SN/VTA degeneration mainly affected reward sensitivity, then both reward conditions in the task (GW and NGW) would be equally affected, rather than the pattern we observe of better performance in one condition (NGW) at the expense of the other (GW) in individuals with greater SN/VTA integrity. We consider this ability to acquire competing responses for rewards as a marker of flexible learning, although we acknowledge this does not translate to overall higher performance levels but rather a more even performance across the different contingencies of the task. Future studies relating midbrain structural integrity to other behavioural indices of flexibility, such as reversal learning, could help to further address the nature of this relationship.

In addition to the SN/VTA, the other structure implicated in modulating NGW performance in older adults was the STN. The STN is a biconvex structure that lies superior to the SN/VTA (Dormont D et al., 2004). Along with other basal ganglia structures, it too is innervated by dopaminergic fibres from the SN/VTA (Hamani C et al., 2004). The STN plays a critical role in action inhibition by relaying a stopping signal (Aron AR and RA Poldrack, 2006) (Frank MJ et al., 2007) (Fleming SM et al., 2010). This inhibitory network depends on interactions between the STN, inferior frontal gyrus and supplementary motor area (Coxon JP et al., 2012) (Duann J-R et al., 2009) (Aron AR et al., 2007; Aron AR and RA Poldrack, 2006) (Jahfari S et al., 2011; Swann NC et al., 2012). Previous work by our own group using the same go/no-go task has shown that inferior frontal gyrus activity is associated with no-go learning and successful instrumental control (Guitart-Masip M, QJM Huys *et al.*, 2012). The current structural SN/VTA and STN findings are therefore compatible with a literature relating functional activity in the post-synaptic targets of midbrain nuclei and their related circuits to both response inhibition and instrumental learning. It is also notable that our VBQ analysis localised NGW learning to structural integrity of the right SN/VTA and STN since inhibitory processing has been reported to evoke a right-lateralised network (Aron AR et al., 2003; Coxon JP *et al.*, 2012; Garavan H et al., 1999; Zheng D et al., 2008).

An important consideration in this study was behavioural and structural differences between young and older adults. Although overall patterns of performance were similar in young and older adults (as shown in **Fig. 2A & Fig. 2B**), some differences emerged which we speculate are linked to age-related neural differences. At a group level, older adults with the lowest MT values of SN/VTA displayed a behavioural inflexibility particularly for rewards. These same adults had significantly lower MT values of SN/VTA than young adults, which might mean they had age-related degeneration of the SN/VTA. Although significantly lower overall performance was observed in the older group, performance in these older individuals was markedly similar to young adults who were unable to learn this task in terms of the observed action by valence interaction during learning. In contrast, performance in older adults with similar midbrain integrity to young adults resembled performance seen in young adult ‘learners’. However it was notable that these older adults who learned to overcome pre-potent response biases did so at the cost of overall task performance. This trade-off between instrumental and Pavlovian systems was not evident in young adults who successfully instrumentally learned. One possible explanation for this is the involvement of other brain regions in young adults performing this task. For example, it has been shown that young adults who are able to instrumentally learn in this task show heightened activity in the inferior frontal gyrus (Guitart-Masip M, QJM Huys *et al.*, 2012). Future studies designed to directly test age-differences in this structure-function relationship could elucidate this further.

An advantage of our study was the use of high quality MT images to accurately identify the SN/VTA and R2* images to define the STN. The MT contrast is particularly suited to visualising brainstem structures as it provides better grey/white matter contrast than the standard T1w MRI contrast (Helms G *et al.*, 2009). MT measures macromolecule concentration and thus reflect the properties of bound protons in structures such as myelin (Tofts P, 2003), axons (Klistorner A et al., 2011), cell membrane proteins and phospholipids (Bruno SD et al., 2004) (Wolff SD and RS Balaban, 1989). Moreover, reduced MT in the SN/VTA has been described in Parkinson’s disease and is proposed to reflect the loss of dopamine neurons (Eckert T *et al.*, 2004) (Tambasco N *et al.*, 2011). We found that some but not all older adults had lower structural integrity of the SN/VTA than young adults. This suggests inter-individual variability of SN/VTA structural integrity amongst older adults and possibly relates to variable dopamine decline as a function of age, although we acknowledge that the exact pathology underlying alterations in the MT signal in normal aging remains unknown. Future studies combining MT imaging with other imaging modalities (e.g. Positron Emission Tomography) and histological evidence will help to provide greater insight into the interpretation of MT values of dopaminergic brainstem structures.

In summary, the new perspective highlighted here is that individual differences of SN/VTA integrity contribute to learning flexibility by allowing older individuals to overcome response biases. In contrast, structural integrity of SN/VTA did not predict instrumental learning in young adults, suggesting that instrumental learning in older age is sensitive to structural changes of the dopaminergic midbrain.

**Acknowledgments**

This work was supported by the Wellcome Trust (grant numbers WT088286MA awarded to RC, 078865/Z/05/Z awarded to RD and core funding for The Wellcome Trust Centre for Neuroimaging 091593/Z/10/Z). The authors would like to thank Professor Stephen Jackson for assistance with recruitment, Nikolaus Weiskopf, Antoine Lutti and John Ashburner for discussions pertaining to the imaging analysis and anonymous reviewers for their valuable comments.

**Financial Disclosures**

 All authors report no biomedical financial disclosures or potential conflicts of interest.

**References**

Abler B, Walter H, Erk S, Kammerer H, Spitzer M (2006) Prediction error as a linear function of reward probability is coded in human nucleus accumbens. Neuroimage 31: 790-795.

Aizenstein HJ, Butters MA, Clark KA, Figurski JL, Andrew Stenger V, Nebes RD, Reynolds Iii CF, Carter CS (2006) Prefrontal and striatal activation in elderly subjects during concurrent implicit and explicit sequence learning. Neurobiology of Aging 27: 741-751.

Aron AR, Behrens TE, Smith S, Frank MJ, Poldrack RA (2007) Triangulating a Cognitive Control Network Using Diffusion-Weighted Magnetic Resonance Imaging (MRI) and Functional MRI. The Journal of Neuroscience 27: 3743-3752.

Aron AR, Fletcher PC, Bullmore ET, Sahakian BJ, Robbins TW (2003) Stop-signal inhibition disrupted by damage to right inferior frontal gyrus in humans. Nat Neurosci 6: 115-116.

Aron AR, Poldrack RA (2006) Cortical and Subcortical Contributions to Stop Signal Response Inhibition: Role of the Subthalamic Nucleus. The Journal of Neuroscience 26: 2424-2433.

Ashburner J (2007) A fast diffeomorphic image registration algorithm. Neuroimage 38: 95-113.

Ashburner J, Friston KJ (2005) Unified segmentation. Neuroimage 26: 839-851.

Bäckman L, Nyberg L, Lindenberger U, Li S-C, Farde L (2006) The correlative triad among aging, dopamine, and cognition: Current status and future prospects. Neuroscience & Biobehavioral Reviews 30: 791-807.

Bruno SD, Barker GJ, Cercignani M, Symms M, Ron MA (2004) A study of bipolar disorder using magnetization transfer imaging and voxel-based morphometry. Brain 127: 2433-2440.

Cloninger CR (1987) The tridimensional personality questionnaire. Version IV. St Louis, MO: Department of Psychiatry, Washington University School of Medicine.

Coxon JP, Van Impe A, Wenderoth N, Swinnen SP (2012) Aging and Inhibitory Control of Action: Cortico-Subthalamic Connection Strength Predicts Stopping Performance. The Journal of Neuroscience 32: 8401-8412.

Dayan P, Niv Y, Seymour B, D. Daw N (2006) The misbehavior of value and the discipline of the will. Neural Networks 19: 1153-1160.

Dickinson A, & Balleine, B (2002) The role of learning in the operation of motivational systems. New York: John Wiley & Sons.

Dormont D, Ricciardi KG, Tande D, Parain K, Menuel C, Galanaud D, Navarro S, Cornu P, Agid Y, Yelnik J (2004) Is the Subthalamic Nucleus Hypointense on T2-Weighted Images? A Correlation Study Using MR Imaging and Stereotactic Atlas Data. AJNR Am J Neuroradiol 25: 1516-1523.

Draganski B, Ashburner J, Hutton C, Kherif F, Frackowiak RSJ, Helms G, Weiskopf N (2011) Regional specificity of MRI contrast parameter changes in normal ageing revealed by voxel-based quantification (VBQ). Neuroimage 55: 1423-1434.

Duann J-R, Ide JS, Luo X, Li C-sR (2009) Functional Connectivity Delineates Distinct Roles of the Inferior Frontal Cortex and Presupplementary Motor Area in Stop Signal Inhibition. The Journal of Neuroscience 29: 10171-10179.

Düzel S, Schutze H, Stallforth S, Kaufmann J, Bodammer N, Bunzeck N, Munte TF, Lindenberger U, Heinze HJ, Duzel E (2008) A close relationship between verbal memory and SN/VTA integrity in young and older adults. Neuropsychologia 46: 3042-3052.

Duzel E, Bunzeck N, Guitart-Masip M, Duzel S (2010) NOvelty-related Motivation of Anticipation and exploration by Dopamine (NOMAD): Implications for healthy aging. Neuroscience & Biobehavioral Reviews 34: 660-669.

Eckert T, Sailer M, Kaufmann J, Schrader C, Peschel T, Bodammer N, Heinze H-J, Schoenfeld MA (2004) Differentiation of idiopathic Parkinson's disease, multiple system atrophy, progressive supranuclear palsy, and healthy controls using magnetization transfer imaging. Neuroimage 21: 229-235.

Fearnley JM, Lees AJ (1991) Ageing and Parkinson's Disease: substantia nigra regional selectivity. Brain 114: 2283-2301.

Fera F, Weickert TW, Goldberg TE, Tessitore A, Hariri A, Das S, Lee S, Zoltick B, Meeter M, Myers CE, Gluck MA, Weinberger DR, Mattay VS (2005) Neural Mechanisms Underlying Probabilistic Category Learning in Normal Aging. The Journal of Neuroscience 25: 11340-11348.

Fleming SM, Thomas CL, Dolan RJ (2010) Overcoming status quo bias in the human brain. Proceedings of the National Academy of Sciences 107: 6005-6009.

Frank MJ, Samanta J, Moustafa AA, Sherman SJ (2007) Hold Your Horses: Impulsivity, Deep Brain Stimulation, and Medication in Parkinsonism. Science 318: 1309-1312.

Frank MJ, Seeberger LC, O'Reilly RC (2004) By Carrot or by Stick: Cognitive Reinforcement Learning in Parkinsonism. Science 306: 1940-1943.

Garavan H, Ross TJ, Stein EA (1999) Right hemispheric dominance of inhibitory control: An event-related functional MRI study. Proceedings of the National Academy of Sciences 96: 8301-8306.

Gray JA, and McNaughton, M. (2000) The neuropsychology of anxiety: an inquiry into the function of the septohippocampal system: Oxford University Press.

Guitart-Masip M, Chowdhury R, Sharot T, Dayan P, Duzel E, Dolan RJ (2012) Action controls dopaminergic enhancement of reward representations. Proceedings of the National Academy of Sciences 109: 7511-7516.

Guitart-Masip M, Huys QJM, Fuentemilla L, Dayan P, Duzel E, Dolan RJ (2012) Go and no-go learning in reward and punishment: Interactions between affect and effect. Neuroimage 62: 154-166.

Hamani C, Saint-Cyr JA, Fraser J, Kaplitt M, Lozano AM (2004) The subthalamic nucleus in the context of movement disorders. Brain 127: 4-20.

Helms G, Dathe H, Kallenberg K, Dechent P (2008) High-resolution maps of magnetization transfer with inherent correction for RF inhomogeneity and T1 relaxation obtained from 3D FLASH MRI. Magnetic Resonance in Medicine 60: 1396-1407.

Helms G, Draganski B, Frackowiak R, Ashburner J, Weiskopf N (2009) Improved segmentation of deep brain grey matter structures using magnetization transfer (MT) parameter maps. Neuroimage 47: 194-198.

Jahfari S, Waldorp L, van den Wildenberg WPM, Scholte HS, Ridderinkhof KR, Forstmann BU (2011) Effective Connectivity Reveals Important Roles for Both the Hyperdirect (Fronto-Subthalamic) and the Indirect (Fronto-Striatal-Pallidal) Fronto-Basal Ganglia Pathways during Response Inhibition. The Journal of Neuroscience 31: 6891-6899.

Kakade S, Dayan P (2002) Dopamine: generalization and bonuses. Neural Networks 15: 549-559.

Klistorner A, Chaganti J, Garrick R, Moffat K, Yiannikas C (2011) Magnetisation transfer ratio in optic neuritis is associated with axonal loss, but not with demyelination. Neuroimage 56: 21-26.

Lambert C, Zrinzo L, Nagy Z, Lutti A, Hariz M, Foltynie T, Draganski B, Ashburner J, Frackowiak R (2012) Confirmation of functional zones within the human subthalamic nucleus: Patterns of connectivity and sub-parcellation using diffusion weighted imaging. Neuroimage 60: 83-94.

Lisman J, Grace AA, Duzel E (2011) A neoHebbian framework for episodic memory; role of dopamine-dependent late LTP. Trends in Neurosciences 34: 536-547.

Lutti A, Hutton C, Finsterbusch J, Helms G, Weiskopf N (2010) Optimization and validation of methods for mapping of the radiofrequency transmit field at 3T. Magnetic Resonance in Medicine 64: 229-238.

Martin WRW (2009) Quantitative estimation of regional brain iron with magnetic resonance imaging. Parkinsonism & Related Disorders 15: S215-S218.

Martin WRW, Wieler M, Gee M (2008) Midbrain iron content in early Parkinson disease. Neurology 70: 1411-1417.

Mell T, Wartenburger I, Marschner A, Villringer A, Reischies FM, Heekeren HR (2009) Altered function of ventral striatum during reward-based decision making in old age. Frontiers in Human Neuroscience 3.

Niv Y, Daw N, Joel D, Dayan P (2007) Tonic dopamine: opportunity costs and the control of response vigor. Psychopharmacology 191: 507-520.

O'Doherty J, Dayan P, Schultz J, Deichmann R, Friston K, Dolan RJ (2004) Dissociable Roles of Ventral and Dorsal Striatum in Instrumental Conditioning. Science 304: 452-454.

Rorden C BM (2000) Stereotaxic display of brain lesions. Behavioral Neurology 12: 191-200.

Salamone JD, Correa M, Mingote SM, Weber SM (2005) Beyond the reward hypothesis: alternative functions of nucleus accumbens dopamine. Current Opinion in Pharmacology 5: 34-41.

Samanez-Larkin GR, Kuhnen CM, Yoo DJ, Knutson B (2010) Variability in Nucleus Accumbens Activity Mediates Age-Related Suboptimal Financial Risk Taking. J Neurosci 30: 1426-1434.

Schultz W, Dayan P, Montague PR (1997) A neural substrate of prediction and reward. Science 275: 1593-1599.

Swann NC, Cai W, Conner CR, Pieters TA, Claffey MP, George JS, Aron AR, Tandon N (2012) Roles for the pre-supplementary motor area and the right inferior frontal gyrus in stopping action: Electrophysiological responses and functional and structural connectivity. Neuroimage 59: 2860-2870.

Tambasco N, Belcastro V, Sarchielli P, Floridi P, Pierguidi L, Menichetti C, Castrioto A, Chiarini P, Parnetti L, Eusebi P, Calabresi P, Rossi A (2011) A magnetization transfer study of mild and advanced Parkinson’s disease. European Journal of Neurology 18: 471-477.

Tofts P (2003) Quantitative MRI of the Brain: Measuring Changes Caused by Disease Wiley.

Vaillancourt DE, Spraker MB, Prodoehl J, Zhou XJ, Little DM (2012) Effects of aging on the ventral and dorsal substantia nigra using diffusion tensor imaging. Neurobiology of Aging 33: 35-42.

Wolff SD, Balaban RS (1989) Magnetization transfer contrast (MTC) and tissue water proton relaxation in vivo. Magnetic Resonance in Medicine 10: 135-144.

Yushkevich PA, Piven J, Hazlett HC, Smith RG, Ho S, Gee JC, Gerig G (2006) User-guided 3D active contour segmentation of anatomical structures: Significantly improved efficiency and reliability. Neuroimage 31: 1116-1128.

Zald DH, Cowan RL, Riccardi P, Baldwin RM, Ansari MS, Li R, Shelby ES, Smith CE, McHugo M, Kessler RM (2008) Midbrain Dopamine Receptor Availability Is Inversely Associated with Novelty-Seeking Traits in Humans. The Journal of Neuroscience 28: 14372-14378.

Zheng D, Oka T, Bokura H, Yamaguchi S (2008) The Key Locus of Common Response Inhibition Network for No-go and Stop Signals. Journal of Cognitive Neuroscience 20: 1434-1442.

**Table 1: Multiple regression results for each predictor variable for no-go to win performance.** The magnetization transfer (MT) value of the SN/VTA was the only significant contributing variable to no-go to win performance in each model. Vol = SN/VTA volume.

|  | **predictor variable** | **β** | **p** |
| --- | --- | --- | --- |
| **Model 1** | T1 | -0.07 | 0.66 |
|  | R2* | 0.08 | 0.59 |
|  | age | 0.20 | 0.18 |
|  | vol | 0.26 | 0.10 |
|  | MT | 0.38 | 0.02 |
| **Model 2** | R2* | 0.08 | 0.57 |
|  | age | 0.20 | 0.19 |
|  | vol | 0.24 | 0.10 |
|  | MT | 0.41 | 0.07 |
| **Model 3** | age | 0.22 | 0.12 |
|  | vol | 0.24 | 0.10 |
|  | MT | 0.40 | 0.007 |
| **Model 4** | vol | 0.21 | 0.17 |
|  | MT | 0.40 | 0.01 |
| **Model 5** | MT | 0.46 | 0.002 |

**Table 2: Voxel-based quantification results for no-go to win positive correlation with grey matter MT images.**

Peak level results are shown for all clusters greater than 10 voxels, p-value <0.001 uncorrected at the whole brain level. SN/VTA = substantia nigra/ventral tegmental area; STN = subthalamic nucleus

| Region | No. voxels | MNI co-ordinates | | | T | Z |
| --- | --- | --- | --- | --- | --- | --- |
|  |  | X (mm) | Y (mm) | Z (mm) |  |  |
| right SN/VTA & STN | 33 | 9 | -16.5 | -7.5 | 3.73 | 3.39 |
| left cerebellum | 16 | -37.5 | -64.5 | -39 | 3.57 | 3.27 |
| left putamen | 13 | -27 | 3 | -4.5 | 3.51 | 3.22 |

**Figure 1: Probabilistic monetary go/no-go task**

**Figure 2: Go/no-go task performance in older and young adults**

(2A, left): Older participants (n = 42) had an asymmetry in action-valence learning, such that they were better at learning active choices for a reward (‘go to win’) than to avoid punishment (‘go to avoid losing’), whereas they were better at learning passive choices to avoid punishment (‘no-go to avoid losing’) than for reward (‘no-go to win’).

(2A, right): Older adults began the task with a bias towards choosing an action (‘go’). Learning occurred in all conditions over the course of the task.

(2B, left & right) A similar overall pattern of behaviour was evident in 47 young adults.

(2C): A subgroup of 12 older individuals with higher SN/VTA integrity (‘high MT’) could overcome response biases to acquire competing responses for reward, compared to a subgroup of 12 older adults with lower SN/VTA integrity (‘low MT’).

(2D): This ‘low MT’ subgroup of older adults (n = 12) had significantly lower MT values of SN/VTA than 12 young adults whereas the ‘high MT’ subgroup of older adults had similar MT values to young adults. Note the young group here is a different set of participants from those whose behaviour is shown in 2B.

Error bars represent ±1 SEM. * 0.01<p<0.05, ** p<0.01, *** p<0.0005.

**Figure 3: Higher no-go to win performance is associated with higher structural integrity of SN/VTA and STN**

(3A): Region-of-interest analysis of the SN/VTA (single subject single slice illustration of the bilateral SN/VTA ROI, blue; see Supplementary Fig. 1 also). Scatter plot (where each dot represents an individual) shows older individuals with higher SN/VTA integrity, indexed by higher magnetization transfer (MT) values, performed better in the no-go to win condition of the task.

(3B): An independent whole-brain voxel-based analysis of MT maps confirmed the association between higher MT values and no-go to win learning, localising to a region overlapping with the right SN/VTA and right STN. Displayed on group-averaged MT image, uncorrected threshold p <0.001.

**Figure 4: Relationship between SN/VTA structural integrity and flexibility of instrumental learning**

(4A): Higher SN/VTA integrity (indexed by magnetization transfer values of SN/VTA) in older adults correlated with both a reduced action bias and reduced interaction between action and valence learning (n = 42).

(4B): No correlation between SN/VTA integrity and the action bias or the action by valence interaction in young adults (n = 30).

Scatter plots, where each dot represents an individual. GW = go to win; GAL = go to avoid losing; NGW = no-go to win; NGAL = no-go to avoid losing
